# Measuring sustainable tourism with online platform data

**DOI:** 10.1140/epjds/s13688-022-00354-6

**Published:** 2022-07-18

**Authors:** Felix J. Hoffmann, Fabian Braesemann, Timm Teubner

**Affiliations:** 1 Trust in Digital Services, Technische Universität Berlin, 10623 Berlin, Germany; 2 Oxford Internet Institute, University of Oxford, OX1 3JS Oxford, UK; 3 Datenwissenschaftliche Gesellschaft Berlin, 10117 Berlin, Germany

**Keywords:** Sustainable tourism, Platform data, TripAdvisor, Nowcasting, Imbalanced classification, Supervised learning

## Abstract

Sustainability in tourism is a topic of global relevance, finding multiple mentions in the United Nations Sustainable Development Goals. The complex task of balancing tourism’s economic, environmental, and social effects requires detailed and up-to-date data. This paper investigates whether online platform data can be employed as an alternative data source in sustainable tourism statistics. Using a web-scraped dataset from a large online tourism platform, a sustainability label for accommodations can be predicted reasonably well with machine learning techniques. The algorithmic prediction of accommodations’ sustainability using online data can provide a cost-effective and accurate measure that allows to track developments of tourism sustainability across the globe with high spatial and temporal granularity.

**Supplementary Information:**

The online version contains supplementary material available at 10.1140/epjds/s13688-022-00354-6.

## Introduction

The tourism industry is of tremendous economic relevance, accounting for an estimated 10% of global GDP in the years before the Covid-19 pandemic [1]. Though strongly affected by restrictions and other uncertainties in international travel, the sector is expected to resume growth and fully recover throughout the coming years [2]. Tourism is also of high importance for economic development, which is underlined by its inclusion in the United Nations’ Sustainable Development Goals (SDGs), where it is directly mentioned in three of the 17 goals [3]. Between 2008 and 2018, the relative importance of tourism for the respective country’s GDP increased in 43 out of 70 countries that report to the UN [4]. At the same time, it has been criticized to have adverse environmental and social effects [5], causing 8% of the global carbon emissions in 2013 [6]. To balance the economic, environmental, and social impacts of tourism, the relevance of sustainable tourism becomes evident [7]. In order to monitor and manage tourism in view of sustainability, granular and accurate spatio-temporal data is needed. There is a growing number of indicator frameworks for the tourism sector that aim to measure sustainability, with the majority of successfully implemented projects focusing on the European market. Current data collection methods, however, are often costly and yield piecemeal results. Ideally, improvements would allow for a cost-efficient implementation in both high income and developing countries, where tourism is growing faster than in more mature markets [8]. In the past, data collection could often be improved by means of tapping into alternative data sources. Examples include the assessment of the digital gender gap based on social network data or the assessment of poverty using mobile phone records [9, 10]. Besides lowering the cost of data collection, such approaches allow for the calculation of indicators in near real-time, rather than relying on year-long cycles.

The present paper expands on such approaches by exploring the feasibility of using online platform data to assess the sustainability of tourism throughout Europe. Specifically, we pose the following research question:

*Research Question*: Can statistical learning techniques using data from an online tourism platform predict tourist accommodations as sustainable, as indicated by a sustainability label?

We use a machine learning approach on online platform data alone to answer the research question. Thus, our study is different from others that discuss rule-based classification systems used by traditional sustainability labels. These labels require detailed information about waste, water use, and other factors to determine an accommodations’ level of sustainability. While highly accurate and true to the causal relationships of sustainability, the corresponding data collection procedures are expensive and not feasible quickly at scale. The classifiers introduced below rely on correlated but not necessarily causal factors. They can hence not fully replace the physical measurement of factors determining sustainability. Instead, the models’ wide applicability and low cost of calculation can serve to complement existing labels, allow for *nowcasting* of sustainability indicators, and increase the geographical coverage of such indicators.

The analysis is based on a unique dataset of *TripAdvisor.com* accommodations and the platform’s *GreenLeader* award. We contribute to the literature in two ways. First, we identify and outline systematic differences between award-holding and non-holding accommodations using public platform data. Secondly, making use of supervised machine learning techniques, we identify sustainable accommodations with reasonable accuracy also in regions that have not implemented the platform’s GreenLeader award. In doing so, we show that large-scale monitoring of sustainable tourism using online platform data in near real-time is feasible. The approach presented here provides a cost-effective and accurate measure with high spatial and temporal granularity, which could be rolled out to track sustainable tourism across the globe.

The remainder of the paper is structured as follows. Section 2 provides an overview of related work and past projects making use of alternative data sources for development statistics in general, and for the assessment of sustainable tourism in particular. Following this, Sect. 3 introduces our methodology, the data set, as well as criteria for model evaluation. Next, Sect. 4 presents our results. In Sect. 5, we then discuss our findings in view of practical and theoretical implications and conclude the paper with limitations and suggestions for further research.

## Background and related work

### Measuring sustainability in tourism

The majority of current sustainability practices in tourism result from regulation and economic incentives rather than intrinsic motivation [11]. Accordingly, policy makers need to define and monitor sustainability in tourism to achieve change. A number of frameworks aim to supply this information by means of indicators (which represent a core element of development research and a central pillar of the SDGs). Next to the UN’s SDGs, tourism-specific indicators were devised by, among others, the World Tourism Organization (UNWTO),^1^ the Global Sustainable Tourism Council (GSTC),^2^ the European Commission,^3^ and the European Environmental Agency (EEA).^4^ Tourism finds direct mention in Goal 8 (‘Decent work and economic growth’), Goal 12 (‘Responsible consumption and production’), and Goal 14 (‘Life below water’) [3]. Note that the use of indicators for the measurement of sustainability in tourism dates back almost three decades, when the World Tourism Organization began to promote their use for policy-making and destination management [12]. Today, the Global Sustainable Tourism Council sets a widely used and accepted standard for sustainability of private companies in tourism based on performance indicators [13]. Moreover, the European Commission first published its ‘European Tourism Indicator System’ (ETIS) in 2013 (with several revisions in the subsequent years). Building on 27 core and 40 optional indicators, ETIS provides the most detailed approach to measure sustainable tourism [14]. At the same time, the EEA has developed a ‘Tourism and Environment Reporting Mechanism’ (TOUERM), monitoring the environmental impact of tourism similar to other industries. Note that the nine TOUERM indicators are similar/overlapping with those of the ETIS framework.

### Sustainability labels

While the aforementioned indicators are mostly geared towards policy making, sustainability labels also serve as a source of information for consumers. Naturally, these labels can also be used to gather information about the state of sustainability in a region or country. Sustainability labels are deemed a suitable means to facilitate ecological progress, especially with regard to clean water and energy, sustainable consumption, and climate protection [11]. Consequently, such labels are considered both by the ETIS and TOUERM frameworks. ETIS indicator A.2.1 can thus be used to gather information relevant for policymakers while relying on third parties’ assessments. It is important to note that there is great variety of sustainability labels, oftentimes leaving consumers left to wonder about their exact meaning, the applied standards, as well as their credibility in view of control mechanisms and enforcement [15].

Beyond institutional labels such as the EU Ecolabel (introduced by the European Commission to highlight low waste, energy efficiency and other sustainability factors [16], online platforms have introduced indicative labels as well. TripAdvisor’s GreenLeader award, for instance, was introduced in the US in 2013 and has been consistently expanded to other countries. Touristic accommodations interested in the award can apply through a questionnaire and must fulfil several standards to become a ‘GreenLeader’. These standards include, among others, towel re-use, recycling, and green roofing [17]. While institutional labels inherit credibility from the sponsoring institution, it is worth taking a closer look at the GreenLeader scheme: The award was devised in cooperation with the UN Environment Programme and has been critically acclaimed. The German Consumer Association highlights its high standards, independence, and transparency. The potential for widespread application stemming from TripAdvisor’s global presence makes the label a useful point of reference. Award-holding listings are decorated with a visual label on TripAdvisor, incentivising accommodations to apply [18].

### Problems of sustainable tourism indicators

The compilation of indicators for sustainable tourism comes with several difficulties, as outlined in the European Tourism Indicator System (ETIS). While some of the data used in the ETIS is readily available from national statistics offices, it is complemented by additional data from surveys and other sources. Rasoolimanesh et al. (2020), for example, reviewed and assessed 97 academic studies on sustainable tourism in terms of relevance for the SDGs, related governance, stakeholders, and the subjectivity of the indicators [7]. Governance-related indicators were found in less than a quarter of all studies, stressing the importance of strong institutions to push evidence-based decision making. Another identified problem is access to and accuracy of data, which play an important role for implementing robust and evidence-based indicators. The European Commission is aware of the time and cost intensity of this approach and suggests not to collect annual data for all indicators, but rather to rely on three-year cycles [14].

Moreover, data reliability is an issue. Modica et al. (2018) asses the initial implementation of the ETIS in the Sardinian region of Cagliari and found that up to 52% of indicator data was missing [19]. Such issues and questions regarding definitions and measurability have, for example, led to the abandonment of indicator 8.9.2 (‘Proportion of jobs in sustainable tourism out of total tourism jobs’) from the Sustainable Development Goals framework [20]. These issues in data collection exist despite high-quality census data and experienced institutions and researchers in the OECD countries. Moreover, there are only few studies on sustainable tourism indicators outside Europe and North America [7]. This means that the data gap on sustainable tourism is increasing, as tourism industries in the Middle East, North Africa, South Asia, and South-East Asia are growing faster than those in Europe and North America [2]. Scholars in development studies suggest yearly or even quarterly reporting of data instead of relying on multiple-year resolution [21]. With traditional methods of data collection, however, this goal seems unattainable.

### Sustainable tourism indicators and online data

To overcome such limitations, online data, which is often created as a byproduct of digital business operations, may complement existing data sources. For example, search engines’ primary function is not the accumulation of popular search terms and their development over time, but this byproduct offers valuable insights for areas such as disease control [22, 23], unemployment statistics [24, 25], or sales forecasting [26].

Through utilizing the above-mentioned strengths and under careful consideration of the associated risks, the use of big data from online sources can increase the quality of existing development evaluations and allow for assessing previously unmeasured outcomes [27]. To better understand how different research approaches aim to achieve this goal, an overview of research facilitating methods of online data collection and analysis in tourism research and related phenomena is presented in Table 1.

**Table 1:** Tabular overview of empirical approaches to measure sustainable tourism, sustainable development indicators and related phenomena with online platform data

Publication	Main variable	Data Source	Data type	Ground truth	Country/Region	Sample size
Bassolas et al. (2016) [39]	Touristic site attractiveness	Twitter	Geolocated tweets and user home locations	–	Africa, Asia, Europe, North and South America	9.6 million geolocated tweets; 59,000 users’ places of residence
Batista e Silva et al. (2018) [32]	Average daily numbers of overnight tourists	Booking.com; TripAdvisor; Eurostat	Accommodation location and capacity	–	EU-28 (incl. GB)	716,103 establishments
Buning and Lulla (2020) [28]	Spatio-temporal usage patterns of bike shares	Bike fleet GPS; user zip codes	GPS; User data	–	Indianapolis, USA	353,733 individual trips
Falk and Hagsten (2020) [34]	Visitor flows to world heritage sites	Instagram; UNESCO	Number of posts, hashtags	–	Europe, North America	680m Instagram posts for 525 sites
Fatehkia et al. (2020) [42]	Wealth Index at clustered geographic locations	Facebook	Advertisement market audience size estimates	DHS wealth index	India and Philippines	1,205 (Philipines); 28,043 (India)
Gallego and Font (2021) [30]	Air passenger demand forecasts	Skyscanner flight searches (ForwardKeys)	Global air capacity and flight search data	–	Global	5,000m searches and >600m picks
Grybauskas et al. (2021) [44]	Apartment revisions	Property listing sites	Property listings with up to 16 numerical features	–	Vilnius, Lithuania	18,922 listings
Hardy and Arval (2020) [29]	Tourist movements in national park	Mobile app; GNSS	Location data; Demographic survey data	–	Tasmania, Australia	472 tourists (4-14 days, 1 signal/10 seconds)
Kashyap and Verkroost (2021) [43]	LinkedIn GGI = gender gaps along different dimensions	LinkedIn	Advertisement market audience size estimates	Country-level professional gender gaps data from international labour organisation (ILO)	Global, up to 234 countries predicted (depending on level of analyis); Up to 185 in ground truth	460 million users, 165,02 million without missing data
Londoño and Hernandez-Maskivker (2016) [38]	Customer feedback to GreenLeader program	TripAdvisor	Sustainability mention, gender, nationality, hotel category, city, GL level	Review concerning sustainability or not	6 Cities in Europe and North America	572 comments
Mariani and Borghi (2021) [37]	eWOM (presence and depth of discourse)	Booking.com; TripAdvisor	Text (Comments)	–	Americas, Europe	4,12 million TripAdvisor and 1,56 million Booking.com reviews
Mendoza et al. (2019) [41]	Intensity of an earthquake in terms of damages	Twitter	Tweets (text mining)	Earthquake catalogue provided by Seismological Center of Chile	Chile	Initially 825,310 tweets; final sample: 187,317 geo-mapped tweets
Nurmi et al. (2020) [31]	Nights spent by foreign tourists	GDS; Amadeus, Sabre, Galileo	Flight bookings	Official accommodation statistics (Finland)	Finland	58 months × 7 countries
Quattrone et al. (2018) [33]	Number of Airbnb listings per tract (geographical unit based on census data)	Airbnb	Geolocations of listings	–	8 cities in USA	54,681 listings
Serrano et al. (2021) [36]	Attributes important to “green tourists”	Airbnb	Text (Comments)	Actual user assessments	Global, 83 cities	more than 176 million comments
Sun and Paule (2017) [35]	Restaurant and bar rating clusters	Yelp	User ratings from 1-5	–	Pheonix, USA	2578 restaurants, 981 fast food restaurants, 797 bars
Talebi et al. (2021) [40]	Potential to become ecotourism destination	Manually collected geo data	Geo data (slope, elevation, soil texture, vegetation, etc.)	Classification from systemic analysis	Arasbaran, Iran	637 recreational areas

For example, GPS trackers and mobile phone applications can be employed to identify patterns of tourist movements in great detail. In combination with app data, Buning and Lulla (2020) were able to differentiate rental bike use between local users and tourists [28]. Furthermore, segments of tourists that are likely to use specific trails and visit at peak times could be identified via matching app-generated location and demographic survey data [29]. Big data can also help for improved touristic capacity management. For instance, tourist flights, overnight stays, and sightseeing crowds can be analyzed and forecasted using big data. This also holds for air passenger demand based on flight price search/comparison websites [30], as well as for the nights spent at certain destinations by tourists with high spatial resolution [31]. Using booking and travel platform data, the density of touristic stays could be estimated for the area of the European Union and Great Britain [32]. Similarly, the number of Airbnb offers per tract in the United States could be calculated [33]. At the level of single touristic sights, visitor flows to World Heritage sites were successfully estimated using Instagram posts [34]. Focusing on the quality of businesses, geographic clusters of similarly rated restaurants and bars were determined using Yelp review data [35].

Furthermore, text sources such as online customer reviews offer the possibility to analyze tourists’ concerns and preferences in real-time. For example, attributes that are important to environmentally aware tourists have been identified using text mining techniques on Airbnb comments [36]. The presence and depth of environmental discourses can be assessed based on booking and travel platform data [37]. TripAdvisor comments, in particular, have been used to understand sustainable practices introduced by accommodations [38]. Twitter data has been used to assess the attractiveness of popular touristic sites [39]. Advanced analytics can also be applied to traditional data sources, for instance, to assess a destination’s potential for ecotourism using artificial neural networks [40].

In addition, online data has found application in the measurement and nowcasting of several other phenomena. For example, Twitter data has been used to assess damages after earthquakes [41]. The estimated size of Facebook ad audiences was used to calculate a wealth index at high spatial granularity [42]. Professional social network data was used to estimate gender gaps within industries and seniority levels [43]. Lastly, the effect of the Covid-19 pandemic on the property sector was estimated using property listing website data [44].

## Methodology

Having reviewed the literature on approaches to measure sustainable tourism, we now introduce the methodology used for the algorithmic identification of GreenLeader accommodations based on publicly displayed data from TripAdvisor.

After establishing the methodological steps of the analysis in the following paragraphs, we investigate the research question whether statistical learning algorithms are able to reveal systematic differences between online profiles of sustainable and non-sustainable accommodations that allow for predicting the existence of a sustainability label.

### Data collection

Data collection took place in November and December of 2020 via web-scraping. For each of the countries included in the analysis, links to all cities with TripAdvisor listings were extracted from the starting page. To obtain direct links to all listings, the algorithm looped through the city URLs and searched for hotel listings in each city. Using this approach, we collected a total of 260,348 individual accommodation listings from 37 European countries. These include all 27 EU member states as well as England, Northern Ireland, Scotland, Wales, Iceland, Liechtenstein, Monaco, Norway, San Marino, and Switzerland.

TripAdvisor provides a broad range of information about accommodations. There are three main sources of data: Page owners (i. e., the accommodation’s operators), consumers, and other/external websites. Next to basic information such as an accommodation’s name and location, page owners can detail their hotel’s size and class, provide contact information, and publish room features and property amenities. Consumers can rate the accommodation on several quality metrics and give an overall rating. They can further provide written reviews, comments, questions, tips, or upload photos. TripAdvisor supplements this information in two ways. First, the website incorporates information from other webpages (e. g., average prices from other websites such as *Booking.com* and *Opodo*). The platform further calculates a score for the accommodation’s location in the city and counts the number of close-by restaurants and attractions using geographic data from Google. Secondly, TripAdvisor accumulates and publishes background data of commentators such as their chosen language as well as trip times and durations. In addition, the website uses customer feedback to create an accommodation ranking within each city.

From the individual listings’ web pages, we collected a total of 102 features, covering five categories of data: the hotel description, its class and ratings (a), prices and information about the size (b), scores calculated by TripAdvisor about the hotel and its location (c), measures of customer interaction (d), and hotel amenities (e).

These features comprise all readily available numeric variables of a listing as well as its binary and ordinal labels. Of the collected information, 15 features relate to hotel description and ratings (a), six variables inform about price segment and hotel size (b), three features relate to location (c). In addition, customer interaction (d) is included through 16 variables for text and image interactions (reviews, uploaded photos). Please note that these variables refer to the amount of user interaction (i. e. number of photos uploaded, number of reviews). The content of photos or reviews is not analyzed. Finally, 62 features inform about the availability of specific amenities and hotel features (e). A detailed summary and description of all variables is provided in Additional file 1 sections I and II.

After splitting the dataset into observations stemming from countries using the TripAdvisor GreenLeader award and those without, 215,806 labelled listings remain available for classifier training. Of these observations, almost 30% have complete information about all variables of interest; 70% have at least one missing value. The variables with most missing values are hotel class (49% missing), TripAdvisor-generated location scores (34% missing) and the number of available rooms (26% missing). The amount of missing data for these variables is critically large – imputation of missing data is not possible here. To make sure not to introduce any bias due to imputation, we therefore exclude all observations with missing values. This leaves 65,515 complete and labelled observations for model training. Comparisons between the full data set (including missing values) and the final data set are provided in Additional file 1 section III. Furthermore, we excluded four observations that contained erroneous records on the number of rooms available (for details, see Additional file 1 section IV).

### Data processing

We undertook several steps of data pre-processing before further analysis. Some variables had to be transformed to deal with skewness. This allowed for the selection and final application of transformations offering the greatest improvement in classifier performance. As dependent variable, we focus on the TripAdvisor GreenLeader Award as a (binary) proxy for an accommodation’s sustainability. A detailed account of all variables’ distributions is provided in Additional file 1 section V.

### Classification

As the next step, we set out to distinguish sustainable and non-sustainable accommodations. To do so, we employ a grid search of preparatory methods and algorithms to find the models with best predictive performance. In total, we ran 360 models based on 3 dimensionality reduction techniques × 5 resampling approaches × 6 data transformations × 4 classifiers. The models are evaluated using three metrics suitable for imbalanced classification tasks. In the following, the components of the analyzed modelling processes and the applied classification metrics are listed. This allows for an understanding of the grid of methods used. A more detailed overview of the pre-processing techniques used in the grid search is provided in Additional file 1 section VI.

Dimensionality reduction (i. e. principal component analysis) of the input data enables the chosen classifiers to work more efficiently and avoids issues of excess dimensionality. Three options are compared in the grid search: Use of the full dataset, use of the first four, and use of the first eight principal components created from all variables.

Moreover, resampling alleviates issues related to imbalanced data by altering the training dataset to have a more equal distribution of labels. The grid search compares modelling processes without resampling with processes using one of four resampling strategies. These are random oversampling, random undersampling, and *S*ynthetic *M*inority *O*versampling *TE*chnique (*SMOTE*), as well as the combination of SMOTE and undersampling.

Additionally, the grid search considered six types of data transformation to adjust the distribution of the input data. The use of the original data is compared to three straightforward (normalization, standardization and robust scaling) and two distribution-dependent transformations (Yeo-Johnson and Box-Cox).

Finally, four classifiers are compared across the grid: Logistic Regression, Linear Discriminant Analysis, Quadratic Discriminant Analysis, and the Random Forest. More details on the classifiers are provided in Additional file 1 section VII.

The metrics used to compare the modelling processes pay special attention to the imbalanced distribution of class labels. In particular, standard accuracy is not a viable metric in this case since a useless model predicting the majority class label for all observations would score highly (with accuracy equalling the proportion of majority class observations in the data, in our case 96%). Thus, the final metrics chosen for comparison are recall, the F2-measure, and the *R*eceiver *O*perating *C*haracteristic *A*rea *U*nder *C*urve (*ROC AUC*). This choice of metrics reflects the importance of recognizing sustainable accommodations despite the comparatively few available cases in the dataset. All metrics are calculated using tenfold cross-validation.

## Results

The TripAdvisor GreenLeader label is awarded to accommodations that fulfil requirements regarding specific sustainability practices. These accommodations make up 4% of all listings in the sample. Clear standards and thorough checks of the accommodations’ claims make the award a reliable source of information. As a first step of our analysis, we compare the relevant variables conditional on the accommodations’ award status (Additional file 1 section VIII). In the following, we highlight several key findings of this analysis.

### Descriptive statistics

**Figure 1:**
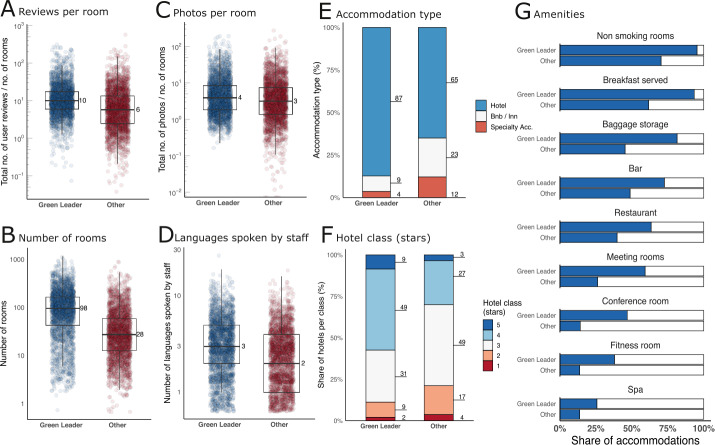
Differences between GreenLeader and other accommodations in TripAdvisor data. (**A**)–(**D**) Distributions of continuous variables: reviews per room, Number of rooms, total number of photos per room and languages spoken by staff in GreenLeader (blue) and other (red) accommodations. (**E**)–(**F**) Proportion of accommodation types and hotel class (stars) in the groups of GreenLeader (left) and other (right) accommodations. (**G**) Proportion of amenities in the groups of GreenLeader (top) and other (bottom) accommodations. GreenLeader accommodations tend to be larger, have more user interactions, are of higher quality, and offer more amenities than other accommodations

There are large differences between GreenLeader accommodations and others in terms of size and type of accommodation as well as user interaction variables (Fig. 1). For example, the median number of reviews received by GreenLeader accommodations (normalized by the number of rooms offered by the accommodation) is 10, while the median is only 6 for other accommodations (Fig. 1(A)). GreenLeader accommodations also tend to be larger, with a median of 96 rooms vs. 28 rooms for other accommodations (Fig. 1(B)). GreenLeaders also differ with regard to the number of uploaded photos (Fig. 1(C)), the number of languages spoken by their staff (Fig. 1(D)), the distribution of accommodation types (Fig. 1(E)), hotel class (Fig. 1(F)), and amenities (Fig. 1(G)). In general, GreenLeader accommodations tend to be larger hotels with more stars, more and diverse amenities, and a higher level of user interaction as measured by reviews and uploaded photos. The variables displayed in Fig. 1 show only a subset of the more than 100 variables that could be derived from the platform data, but they illustrate the differences in publicly available features that appear to be correlated with an accommodation’s sustainability (see Additional file 1 section V for correlation matrices between the GreeanLeader badge and (a) numeric variables, (b) binary variables in the data set). In the following, we illustrate how unsupervised and supervised statistical learning techniques reveal structure in the data to distinguish groups of hotels and other accommodations that show higher or lower shares of sustainability.

### Unsupervised statistical learning

**Figure 2:**
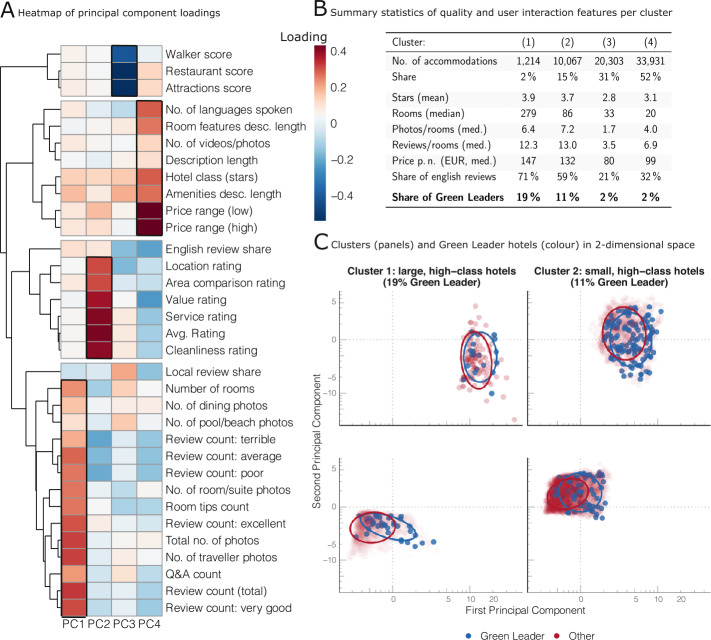
Unsupervised Learning techniques applied to TripAdvisor data. (**A**) Heatmap of principal component loadings of the four main principal components based on dimensionality reduction of the 33 continuous variables in the data set. The algorithm identifies four main dimensions in the data: accommodation size and user interaction (PC1), user rating (PC2), location (PC3), and quality (PC4). (**B**) Summary statistics of four clusters identified by k-means clustering. The accommodations can be grouped according to quality and user interaction variables. The clusters show different proportions of the GreenLeader outcome variable, varying from 2% to 19%. (**C**) Two-dimensional representation (PC1, PC2) of TripAdvisor data (10% sample) grouped in four clusters (panels) and GreenLeader/other accommodations (color). The unsupervised learning algorithms are able to split the data into distinct groups with varying proportions of GreenLeader accommodations

Figure 2 displays the results of dimensionality reduction (principal component analysis) and cluster analysis (k-means) applied to the 33 continuous variables in the data set. The exploratory dimensionality reduction reveals that four components capture a large share of the variation in the data (see Additional file 1 section IX). Figure 2(A) illustrates the loadings of the first four components. We have used hierarchical clustering to identify groups of variables with similar loadings (see dendrogram in Fig. 2(A)). This analysis reveals that the variables tend to group into four clusters, which represent distinct types of information available about each accommodation. The variables Walker score, Restaurant score, and Attractions score all describe the location around the accommodation. A second group of variables describes quality indicators, such as the number of languages spoken by staff, hotel class (stars), and price. Variables related to the user rating (e. g., value, service, and average rating) form a third distinct group, while the fourth cluster describes aspects related to the size of the accommodation (number of rooms, number of reviews). As shown in Fig. 2(A), the data has a structure that can be detected by unsupervised learning algorithms, which reflects interpretable concepts and trust cues known from other domains of the platform economy [45].

In addition to dimensionality reduction, we also use k-means clustering to identify groups of similar accommodations. At this point we want to underline that the cluster analysis is used as an exploratory statistical analysis in this study only. It serves as a way to illustrate that accommodations, which share certain features (among them the sustainability label), tend to co-occur in the data.

Note that many choices on the number of clusters are possible and justifiable (Additional file 1 section X). Here, we used four clusters for separating the data into prototypical groups. Figure 2(B) provides summary statistics of the four groups. Cluster 1 represents a small subset of the data containing only 2% of all accommodations, 19% of which are GreenLeader accommodations. The group is characterized by high quality hotels with many rooms, a lot of user interactions (reviews and photos), many international guests (high share of English language reviews), and high prices. Cluster 2 also contains a disproportionately large share of GreenLeaders, high-quality, and expensive hotels. In contrast to Cluster 1, however, this Cluster’s accommodations are significantly smaller. The largest share of the data is captured by Clusters 3 and 4, which account for 83% of all accommodations but with an average share of only 2% GreenLeader accommodations. Compared to the other two clusters, the accommodations in these groups are substantially less expensive and smaller, they have significantly fewer user interactions, a lower share of English language reviews, and lower quality. The main differences between Cluster 3 and 4 are, again, size and quality: Cluster 3 accommodations have, on average, 50% more rooms than those in Cluster 4, but they score lower on price and quality characteristics (stars), internationality, and user interactions.

In summary, the total dataset can be split into (at least) four groups of hotels: large, high-class hotels in cluster 1, small, high-class hotels in cluster 2, low-price hotels in cluster 3, and other accommodations in cluster 4. Clusters 1 and 2 show the highest share of GreenLeader accommodations.

The cluster differences and the differences between GreenLeader and other accommodations are shown in Fig. 2(C) in the dimensionality-reduced space of the first two principal components. Each panel represents a cluster from the table in Fig. 2(B); circles indicate the position of the majority of the data points of the two accommodation types (GreenLeader vs. non-GreenLeader) in each panel. The plot shows that the different clusters take distinct positions within the two-dimensional space. Accommodations in Cluster 1 – the high-quality hotels with many rooms – score high on the first principal component (which loads heavily on variables related to a hotel’s size) but stretch along the second axis. Similarly, Cluster 2 shows a relatively high loading on the first principal component, but in contrast to Cluster 1, the data tend to show higher values on the second component (reflecting, for instance, better user ratings). Clusters 3 and 4 – the relatively low-price accommodations – both score low on the first component, that is, they represent smaller accommodations. Their main difference is the second principal component: Cluster 3 accommodations seem to be characterized by low user ratings, while Cluster 4 comprises more accommodations with a positive rating. Note, however, that while there are some differences in the positioning within each panel, there is also a large overlap between both groups. The differences are most pronounced in Clusters 3 and 4, which indicates that the GreenLeader accommodations differ more strongly from other accommodations in the realm of lower quality, low-price accommodations.

Overall, the application of the unsupervised learning techniques in this section reveals structures in the data on hotel descriptions, amenities, user ratings, and customer interactions that seem to be correlated with the presence of the GreenLeader sustainability label. We do not make any claims as to whether there exists any causal relationship between these features and the sustainability label; we only observe that they tend to co-occur in the data.

### Classification performance and extrapolation

**Figure 3:**
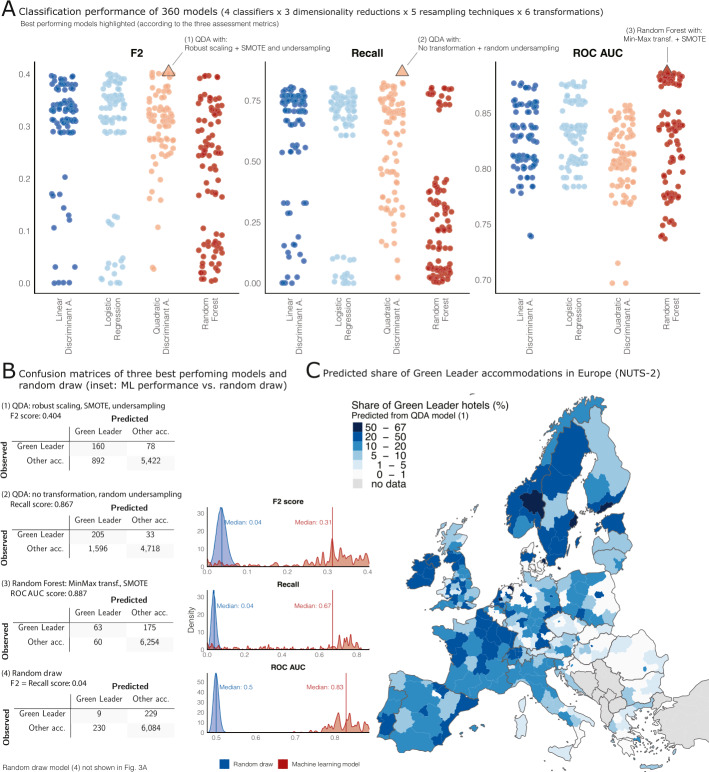
Classification performance and extrapolation. (**A**) Comparison of 360 classification models (90 models per classifier and panel) regarding three performance metrics: F2 score, Recall, and ROC AUC (each dot represents a model). QDA model (1) shows the highest F2 score, QDA model (2) achieves the highest Recall, and Random Forest model (3) the best ROC AUC score. (**B**) Confusion matrices of the three best performing models (1) to (3) according to the performance metrics (top panels) and a random draw model (4) (lowest panel). Inset: performance comparison between machine learning models (red) and 20,000 random draws (blue) according to F2 score, Recall, and ROC AUC. The machine learning models show a significantly better prediction performance than the random draw models. (**C**) Predicted share of GreenLeader accommodations in Europe (NUTS-2) according to the QDA model (1). The model predicts urban centres and several regions in West and North Europe to have the high shares of sustainable tourist accommodations

The aim of the study is to investigate whether algorithmic machine learning algorithms trained on publicly available data can identify and predict the extent of sustainable tourism with high temporal and spatial accuracy. To do so, we tested large sets of models and preprocessing techniques and their combinations. To compare the prediction performance of the models, we report cross-validated average effects on three established metrics: F2 score, Recall, and ROC AUC. In Fig. 3, we provide the main results. Detailed results are provided in Additional file 1 section XI.

The grid search comprised a total of 360 models (i. e., 4 classifiers × 3 dimensionality reduction techniques × 5 resampling methods × 6 data transformations) and resulted in widely varying performances (Fig. 3(A)). Depending on the assessment metric, several model specifications show similar performance. However, many models are not competitive. For example, a large number of Random Forest models score low on the Recall metric.

Fig. 3(A) shows the best performing models according to three assessment matrices: QDA model (1) according to the F2 score (F2 score = 0.404), QDA model (2) according to Recall (Recall = 0.867), and Random Forest model (3) according to the ROC AUC score (ROC AUC = 0.887). To give an intuition into the quality of the prediction, Fig. 3(B) shows the confusion matrices of the cross-validated average prediction accuracy of these models in comparison to a random draw model (4). Please note that each of the listed models uses the same set of variables. They differ with respect to the specification of the full machine learning pipeline, i. e. model choice and preprocessing steps.

High performance on the F2 score as seen for QDA model (1) indicates both, high degrees of precision in correctly identifying non-sustainable hotels and recall of sustainable accommodations. As the latter is at the core of this project, we also look at recall individually. Here, QDA model (2) is able to identify the largest percentage of true GreenLeader listings. Finally, Random Forest model (3) has the highest ability of discriminating between the two classes and hence scores highest in the ROC AUC metric.

From comparing the confusion matrices, but also the overall prediction performance of the different models displayed in Fig. 3(A), it is obvious that there is not one single best choice. This is due to statistical uncertainty, but also because of the fuzziness of the sustainability concept and data used here. Furthermore, as Figs. 1 and 2 show, the data vary in terms of quality, user ratings, location, and interaction metrics. GreenLeader and other accommodations overlap in this regard. Hence, it is not surprising that machine learning models will not be able to perfectly differentiate between the groups.

Nonetheless, it becomes clear that the data allows for the training of statistical learning models that assess the sustainability of touristic accommodations with a level of accuracy far beyond random draw (Fig. 3(B): confusion matrix of model (4) in the lowest panel). The overall predictive capability of the machine learning models trained on the TripAdivsor data is highlighted in the inset of Fig. 3(B). It compares the distributions of the prediction performance of 20,000 random draws using the unconditional probability of 4% GreenLeader accommodations (blue) in the data with the performance of all machine learning models from Fig. 3(A) (red). Since the employed metrics of F2 score, Recall and ROC AUC are less intuitive than simple accuracy, we use random draw as a comparative baseline. The median performance of the machine learning models outperforms random draw substantially: by a factor of 8 with regard to the F2 score, by a factor of 17 with regard to recall, and by a factor of 1.67 with regard to the ROC AUC. In other words, the publicly available TripAdvisor data is informative with respect to the sustainability of touristic accommodations.^5^

To illustrate the granularity of the derived touristic sustainability measure, Fig. 3(C) shows the predicted share of GreenLeader accommodations in the European NUTS-2 regions for countries with and without TripAdvisor’s GreenLeader program. Touristic sustainability follows a particular spatial distribution that seems to be related to the socio-economic structure in Europe. While it is beyond the scope of this study to explain the geography of touristic sustainability in detail, several observations can readily be made. First, the predicted share of GreenLeader accommodations is higher in metropolitan than in rural areas. For example, Berlin, Hamburg, Birmingham, London, Stockholm, Helsinki, Copenhagen, Vienna, Warsaw, Prague, Bratislava, Budapest, Zagreb, Madrid, and Sofia all seem to host much higher share of sustainable accommodations than the surrounding countryside. Moreover, sustainable tourism seems to be more widespread in North and West Europe than in East and South Europe.^6^

In summary, our findings illustrate that the automatized and algorithmic prediction of sustainable tourism indicators is feasible. This can contribute to providing a cost-effective, accurate, and spatially granular assessment and tracking of sustainable tourism over time. The method showcased here can also have positive effects on transparency and thus support informed customer decisions. Moreover, it can help platforms and other organizations to identify sustainable accommodations.

## Discussion

Tourism plays an important role in economic development across the globe and indicators are crucial to understand its development in different regions over time. Though heavily affected by the Covid-19 pandemic, the sector is expected to resume its growth path soon. With it, the environmental and social impacts of tourism will also continue to grow. Measuring and fostering sustainable tourism through effective indicators is thus a topic of global interest. Today, the global use of sustainable tourism indicators is limited by implementation costs and difficulties in data collection. In other areas, the inclusion of alternative data sources has been proven to be beneficial. This paper sets out to test the applicability of an alternative data source for the measurement of sustainable tourism.

### Summary of the results

In collecting and analyzing data from TripAdvisor – one of the globally leading online tourism platforms – we show that it is possible to create a cost-effective, granular, and accurate measure of sustainable tourism based on publicly available online data. We compare differences between touristic accommodations holding TripAdvisor’s GreenLeader award and other accommodations regarding hotel quality metrics, user interaction, user rating, and location features. We conduct a grid search on a total of 360 machine learning pipelines to differentiate sustainable from non-sustainable hotels based on the high-dimensional platform data. The performance of the machine learning models is substantially better in identifying sustainable hotels than the baseline model of unconditional expectation.^7^ In other words, machine learning models trained on online platform data can make a contribution in assessing the state of sustainable tourism in countries and regions as the presence of the sustainability award shows correlation with various other characteristics. Note that some caution is due since these correlates are – in most cases – not formative for the accommodation’s sustainability.

For example, we find that more expansive and larger hotels with more rooms and customer reviews (see Fig. 2(B)) tend to have a higher share of GreenLeader badges. We want to state explicitly that we do not assume such features to be causal for an accommodation’s sustainability. However, both could, nonetheless be related. It might be the case that larger hotels have more resources available to deal with the requirements of sustainability labels. It could also be that larger hotels are more dependent on web traffic from online platforms and therefore invest more resources in obtaining badges from the platform. Furthermore, hotels in the premium segment with a focus on quality and user satisfaction might want to utilise the sustainability label as an additional quality criterion. While such factors are not displayed in the large-scale online platform data, the observable correlations between prices or hotel size and the sustainability label seem to capture such patterns.

The approach presented here reveals factors that correlate with the sustainability label, but it should not be employed to assess individual accommodations’ degree of sustainability based on the correlations alone. However, the approach may well serve to statistically assess countries’ and regions’ degree of sustainable tourism with high temporal and spatial granularity, for example for the purpose of nowcasting sustainability indicators or for extending the geographical coverage of such indicators to places without ‘ground-truth’ data on sustainable tourism.

It is important to highlight that the purpose of the analysis is not in predicting and identifying *individual* hotels as sustainable, but on providing a probabilistic assessment about the distribution of sustainable hotels in a region (as shown in Fig. 3(C)) derived from the prediction model, which uses individual-level data. In that sense, our analysis is similar to medical studies that aim to quantify population-wide health risks. Such assessment consider individual-level risk factors such as age, obesity or nutrition to calculate an estimate of the share of the population being at risk of cardiovascular diseases, but they do not aim to make predictions on the level of individuals patients.

### Theoretical implications for the applicability of big data in tourism research

Past studies have employed text analysis to understand user preferences and discussions [36, 37]. Accommodation-specific data has been used for the estimation of visitor capacities in neighborhoods [32]. In contrast, this paper focuses on the classification of accommodations by sustainability. We add to the literature by utilizing accommodations’ own presentation and associated user interactions to gain information about their sustainability practices. The creation of a large numeric dataset allows for the training of common machine learning algorithms. Using available ground truth data for classifier training, the quality of the analyzed classifiers could be assessed in detail. This approach was followed in prior work where it allowed for comprehensive model assessment using true values and labels [9, 31]. In doing so, we were able to confirm the applicability of travel platform data for use in tourism statistics. In particular, the low cost of data collection and high spatial resolution of the data could be shown. It should be noted that the estimated models do not attempt to create an alternate definition of sustainability through using new causal factors. Instead, the true label is determined trough physical measurements of energy, waste and water. Here, it is estimated using correlated factors available in the online platform data. The legitimacy of using of private company sources for the collection of data for policy making will remain an important open question. Our study helps to underline the potential of the large-scale analysis of online data as a valuable method for research in sustainable tourism, and sustainability studies in general.

### Implications for tourism practitioners and policy makers

Tourism platforms can make use of our findings in multiple ways. Listings, which are predicted to employ sustainability practices but do not (yet) carry the award, can be actively approached, and be made aware of the GreenLeaders program. These accommodations can, in turn, benefit from increased visibility and increase their attractiveness for environmentally conscious consumers. More visibly communicating sustainability efforts could hence become a competitive advantage. This could in turn increase pressure on competing businesses to also invest in sustainable practices.

For platforms, cooperation with researchers and statistics departments is an effective way of underlining environmental and social efforts. Policy makers can benefit from the availability of inexpensive, granular, and up-to-date data. For policy makers in countries with established frameworks for sustainable tourism statistics, the higher frequency and granularity of reporting can offer important supplementary information. Through the comparison with traditional data sources, model accuracy can be monitored, and models can be adjusted where needed. In countries without established frameworks, the proposed methodology can offer estimates when traditional methods of data collection are prohibitively costly to implement, or where important infrastructure is not available.

These estimates can guide policy makers towards initial interventions and allow for detailed monitoring of the associated effects. In relation to existing frameworks of sustainable tourism indicators, the implications are twofold: For the ETIS framework, which collects data on the percentage of accommodations using a voluntary sustainability label under its indicator A.2.1., the described models can offer a remedy for difficulties in data collection for this indicator. For other frameworks, inclusion of the presented indicator can be discussed to create a more complete picture without significantly increasing data collection efforts.

### Practical implications and limitations

It is important to note that the proposed methodology cannot replace accommodation surveys and other statistically robust modes of data collection. It should instead be considered as a complementary source of information or a first estimate when no other data is available. The presented methodology is heavily reliant on the quality of the big data sample. Systematic differences between accommodations listed on TripAdvisor and those that are not should hence be a focal point of further research. Another limitation might be potential confounding factors on the regional level that we could not control for. Our analysis solely uses variables on the level of individual accommendations. For example, it might be that legal requirements or cultural values in some regions affect the share of hotels with a sustainability label.

In addition, omitting incomplete observations may further limit the validity of the training sample and alternatives should be explored in greater detail. Future research should furthermore explore the feasibility of other, freely available data sources. Both, accommodation characteristics and the sustainability label, are taken from TripAdvisor. Characteristics could be collected from a range of other travel platforms. An alternative label for classifier training could be created from other well-established sustainability programs. Although a broad range of classifiers and preparatory steps was compared, other approaches may yet outperform the methods included in the analysis. Lastly, this paper treats sustainability as a binary variable, separating accommodations into those following any sustainability practices and those following none. Additional research could explore whether the degree of sustainability practices, expressed for example by the differentiated TripAdvisor GreenLeader labels from ‘partner’ to ‘platinum’ level, can also be modelled.

On a more general note, the methodology suffers from shortcomings common to all big data approaches. Although new forms of data collection and analysis have filled data gaps and increased our understanding of social, economic, and touristic activity, there are justifiable concerns about the use of such (alternative) data sources. Machine learning methods have the outstanding ability of combining many weak signals into predictions for labels or variables. These signals do not need to be in line with theoretical groundwork and in practice will often not be. For this reason, some algorithmically derived signals would not have been included as relevant explanatory variables in traditional modelling [21]. Algorithmic prediction models also reproduce existing biases in the data. In our case, not all hotels that would fulfil the criteria to obtain a GreenLeader badge might have actually applied for the label – a bias that our model cannot control for. Additionally, the use of big data and machine learning did cause concerns regarding privacy issues and the possibility that algorithms might pick up unethical or discriminatory practices present in historical data sets [46]. However, with the advantage of improved detection of patterns in the data comes the risk of disregarding underlying theory altogether [47].

## Concluding note

This paper set out to analyze whether online platform data can be used to inform about sustainable tourism. Sustainability in tourism describes the goal of balancing economic, social, and environmental factors. The complexity of this goal requires diverse sources of information to monitor progress and inform decision-makers. The corresponding data collection processes offer room for improvement with regards to both cost and frequency of reporting. Research is often focused on Europe, the world’s largest tourism market, but despite freely available national census data and experienced practitioners there are difficulties in the implementation of existing indicator frameworks and the collection of relevant data.

In this paper, we offer an alternative to existing methodologies through the use of travel platform data. In this extended pilot, the platform TripAdvisor was used as the sole source of data. Tourist accommodation data was collected through automated scraping of TripAdvisor listings from 37 European countries. Following several data exploration steps, we developed a supervised learning model for the assessment of accommodations’ sustainability. Ground truth data was sourced from TripAdvisor (where the GreenLeader award is available in 27 of the 37 countries). The final model was chosen from a set of four supervised learning techniques, each building upon combinations of dimensionality reduction, resampling, and data transformation methods. The imbalanced nature of the classification task added difficulty. With less than 4% of training data belonging to the sustainable class, use of the accuracy metric would have been misleading. Model comparison was hence performed using the F2-metric. Recall and the Receiver Operating Characteristic Area Under Curve metric. A classifier using quadratic discriminant analysis was chosen as the final model. Overall, prediction quality was high but not excellent, with all methods struggling to successfully recognize observations from the positive class without significantly increasing the proportion of false positive predictions.

All findings are subject to limitations, the most important being the yet unconfirmed validity of the collected sample for the population of accommodations in each country. This representativeness of the sample and the use of other readily available platform data sources should be the focus of further research.

## Supplementary Information

Below is the link to the electronic supplementary material.

Supplementary information (PDF 6.6 MB)

## Data Availability

The datasets analysed during the current study and code produced for the analysis are available in the author’s GitHub repository: https://github.com/felixjhoffmann/SustainableTourism/.

## Footnotes

1 https://www.unwto.org

2 https://www.gstcouncil.org

3 https://ec.europa.eu

4 https://www.eea.europa.eu

5 In this study, we make the comparison of the performance of our models with a simple random draw model only to illustrate that there is an informative signal contained in the online platform data, which has predictive capacity that can be identified via statistical learning algorithms. We do not want to claim that a random draw model would be the only alternative modelling strategy. In fact, many different types of models can lead to good predictions, as we show by using a set of 360 different models in total.

6 For average sampled and predicted values by country, please refer to Additional file 1 setion XII.

7 The performance of the models is compared to a random draw only for the purpose of illustrating the predictive capacity in the data, see footnote 5.
