# Supplementary material for: Measuring sustainable tourism with online platform data
Source: EPJ Data Sci. 2022 Jul 18;11(1):41. doi: 10.1140/epjds/s13688-022-00354-6 (PMC9289659; doi:10.1140/epjds/s13688-022-00354-6)
Supplement: Supplementary file 1 — Supplementary information (PDF 6.6 MB) [file 13688_2022_354_MOESM1_ESM.pdf]

Measuring Sustainable Tourism with Online Platform Data

SUPPLEMENTARY INFORMATION

Abstract
Sustainability in tourism is a topic of global relevance, finding multiple mentions in the United Nations Sustainable Development Goals. The complex task of balancing tourism's economic, environmental, and social effects requires detailed and up-to-date data. This paper investigates whether online platform data can be employed as an alternative data source in sustainable tourism statistics. Using a web-scraped dataset from a large online tourism platform, a sustainability label for accommodations can be predicted reasonably well with machine learning techniques. The algorithmic prediction of accommodations' sustainability using online data can provide a cost-effective and accurate measure that allows to track the degree of sustainable tourism across the globe with high spatial and temporal granularity.

Keywords
Sustainable Tourism, Platform Data, TripAdvisor, Nowcasting, Imbalanced Classification, Supervised Learning

This Supplementary information contains all methodological details for the full replicability of the results.

All code and raw data are publicly available on GitHub.

Table of Contents

Supplementary Information *3*

Supplementary Information I: Variable location on TripAdvisor page **3**

Supplementary Information II: Overview of variables **7**

Supplementary Information III: Summary statistics for removed incomplete observations .. **17**

Supplementary Information IV: Outlier candidates and adjustments **22**

Supplementary Information V: Variable distributions **26**

Graphical comparison of univariate distributions 29

Correlation between variables 31

Supplementary Information VI: Description of pre-processing techniques used in grid-search **33**

Dimensionality Reduction 33

Sampling Methods 33

Transformations 35

Supplementary Information VII: Description of classifiers used in grid-search **38**

Logistic Regression 38

Linear Discriminant Analysis 39

Quadratic Discriminant Analysis 39

Random Forest Classifier 40

Supplementary Information VIII: Comparison of variable distributions between GreenLeaders and non-GreenLeaders in final sample **42**

Supplementary Information IX: Principal Component Analysis **47**

Supplementary Information X: Clustering Analysis **48**

Scree plots for K-means clustering using four and eight PCs 48

Silhouette plots for K-means clustering of the largest four PCs 50

Silhouette plots for K-means clustering of the largest eight PCs 50

Supplementary Information XI: Classifier performance **51**

Supplementary Information XII: Estimated proportion of GreenLeaders by country **55**

Literature *56*

Supplementary Information

Supplementary Information I: Variable location on TripAdvisor page

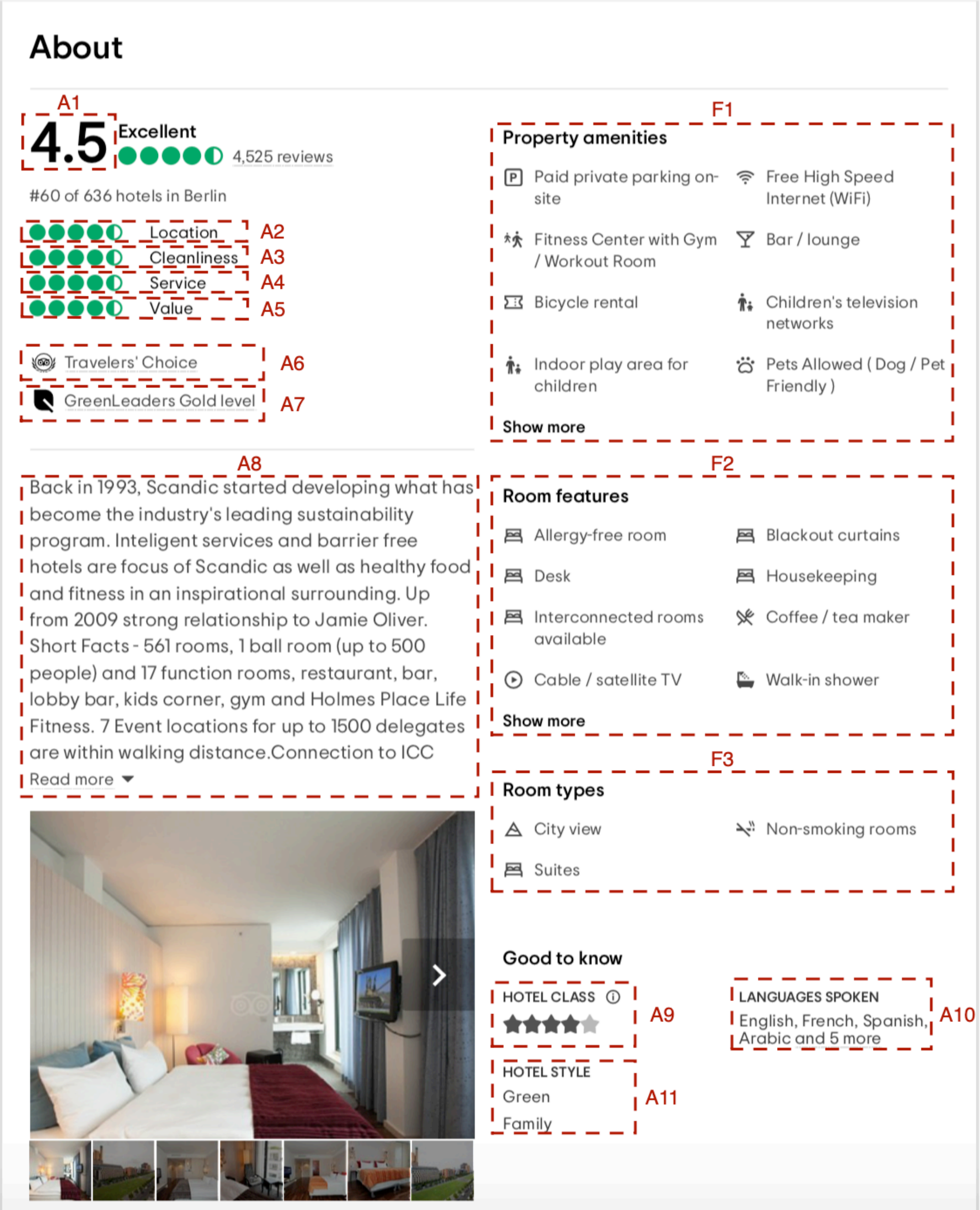

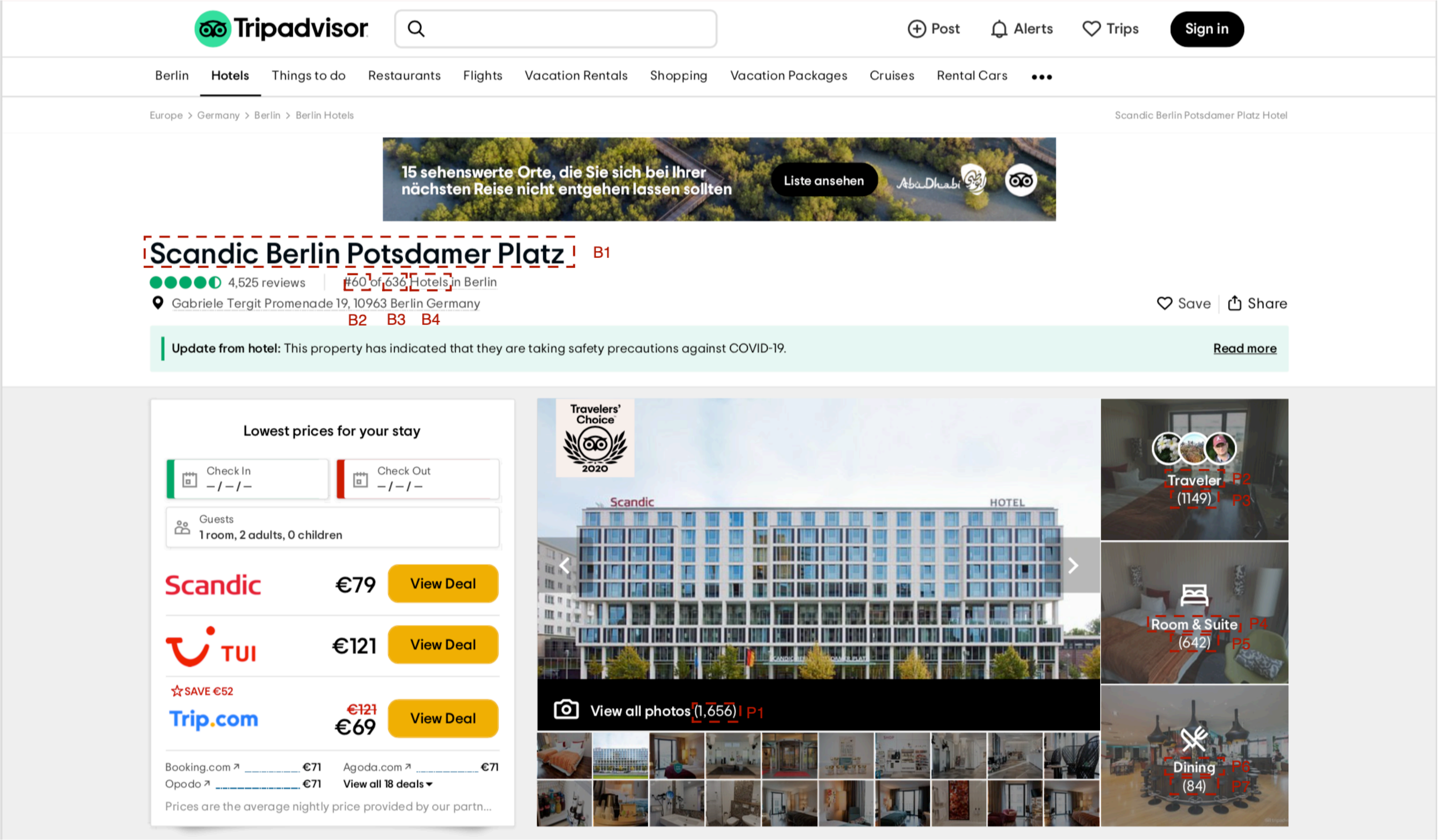

Tripadvisor
Post
Alerts
Trips
Sign in
Berlin
Hotels
Things to do
Restaurants
Flights
Vacation Rentals
Shopping
Vacation Packages
Cruises
Rental Cars
Europe > Germany > Berlin > Berlin Hotels
Scandic Berlin Potsdamer Platz Hotel
15 sehenswerte Orte, die Sie sich bei Ihrer nächsten Reise nicht entgehen lassen sollten
Liste ansehen
Scandic Berlin Potsdamer Platz
B1
4,525 reviews
#60 of 636 Hotels in Berlin
Gabriele Tergit Promenade 19, 10963 Berlin Germany
B2
B3
B4
Save
Share
Update from hotel: This property has indicated that they are taking safety precautions against COVID-19.
Read more
Lowest prices for your stay
Check In
–/–/–
Check Out
–/–/–
Guests
1 room, 2 adults, 0 children
Scandic
€79
View Deal
TUI
€121
View Deal
SAVE €52
Trip.com
€121
€69
View Deal
Booking.com €71
Agoda.com €71
Opodo €71
View all 18 deals
Prices are the average nightly price provided by our partn...
Travelers' Choice 2020
Scandic
HOTEL
View all photos (1,656)
P1
Traveler
(1149)
P2
P3
Room & Suite
(642)
P4
P5
Dining
(84)
P6
P7

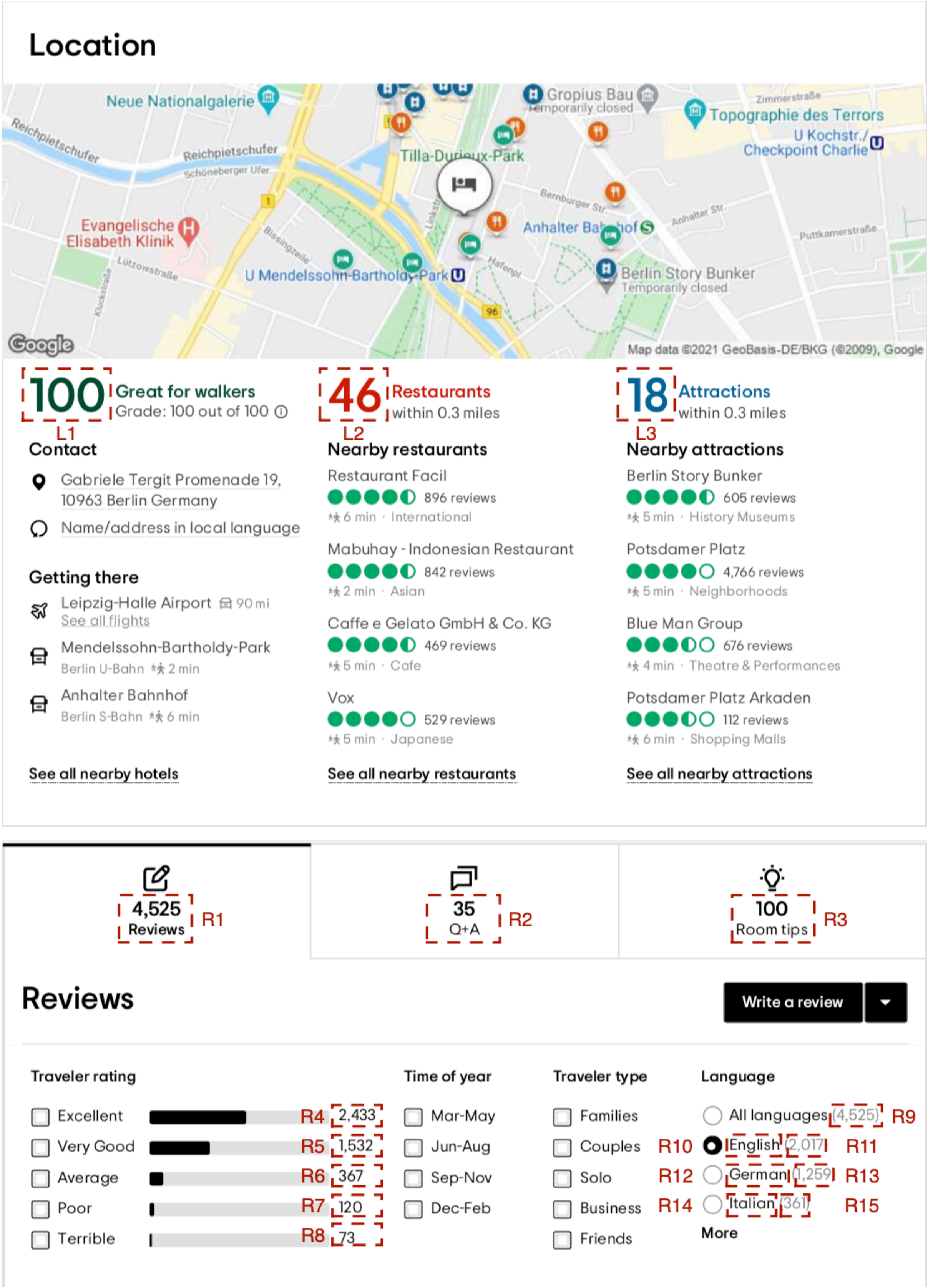
Location
Neue Nationalgalerie
Gropius Bau
Temporarily closed
Topographie des Terrors
U Kochstr./ Checkpoint Charlie
Tilla-Durieux-Park
Evangelische Elisabeth Klinik
U Mendelssohn-Bartholdy-Park
Anhalter Bahnhof
Berlin Story Bunker
Temporarily closed
Map data ©2021 GeoBasis-DE/BKG (©2009), Google
100 Great for walkers
Grade: 100 out of 100
L1
46 Restaurants
within 0.3 miles
L2
18 Attractions
within 0.3 miles
L3
Contact
Gabriele Tergit Promenade 19, 10963 Berlin Germany
Name/address in local language
Getting there
Leipzig-Halle Airport 90 mi
See all flights
Mendelssohn-Bartholdy-Park
Berlin U-Bahn 2 min
Anhalter Bahnhof
Berlin S-Bahn 6 min
See all nearby hotels
Nearby restaurants
Restaurant Facil
896 reviews
6 min · International
Mabuhay - Indonesian Restaurant
842 reviews
2 min · Asian
Caffe e Gelato GmbH & Co. KG
469 reviews
5 min · Cafe
Vox
529 reviews
5 min · Japanese
See all nearby restaurants
Nearby attractions
Berlin Story Bunker
605 reviews
5 min · History Museums
Potsdamer Platz
4,766 reviews
5 min · Neighborhoods
Blue Man Group
676 reviews
4 min · Theatre & Performances
Potsdamer Platz Arkaden
112 reviews
6 min · Shopping Malls
See all nearby attractions
4,525 Reviews
R1
35 Q+A
R2
100 Room tips
R3
Reviews
Write a review
Traveler rating
Excellent R4 2,433
Very Good R5 1,532
Average R6 367
Poor R7 120
Terrible R8 73
Time of year
Mar-May
Jun-Aug
Sep-Nov
Dec-Feb
Traveler type
Families
Couples R10
Solo R12
Business R14
Friends
Language
All languages (4,525) R9
English (2,017) R11
German (1,259) R13
Italian (361) R15
More

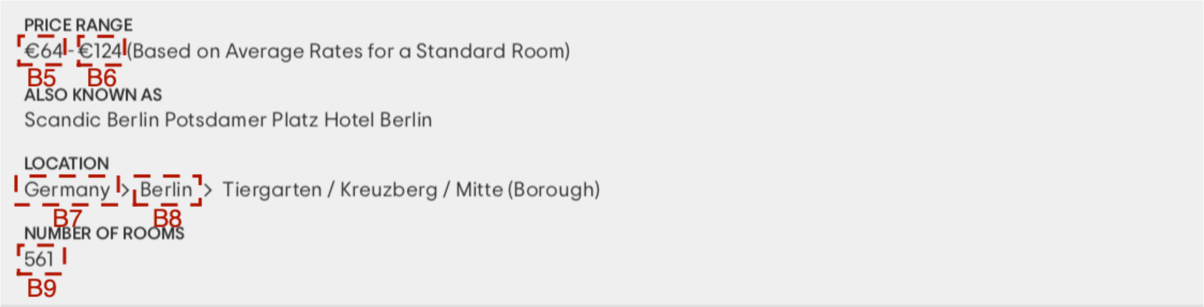
PRICE RANGE
€64 - €124 (Based on Average Rates for a Standard Room)
B5
B6
ALSO KNOWN AS
Scandic Berlin Potsdamer Platz Hotel Berlin
LOCATION
Germany > Berlin > Tiergarten / Kreuzberg / Mitte (Borough)
B7
B8
NUMBER OF ROOMS
561
B9

Supplementary Information II: Overview of variables

In the following, the modification and recoding of all variables is described. Screen captures of the relevant website sections are available in supplementary information I. A detailed summary of modifications can be found in supplementary information II. The *GreenLeader* and *Traveler's Choice* awards are only displayed on a listing's page if they were awarded by TripAdvisor. The variables are binary coded in the final dataset, whereby a listing is assigned the value 0 if no mention of the respective award is made on the page. *GreenLeader* award levels *Partner*, *Bronze*, *Silver*, *Gold*, and *Platinum* are assumed to represent similar characteristics and are recoded to 1 for the *GreenLeader* variable in the final dataset. Similarly, both the *Traveler's Choice* award and the *2020 Traveler's Choice Best of the Best* award are recoded to 1 for the final *Traveler's Choice* variable. The *description* section offers page owners the possibility of describing the accommodation in detail and highlighting unique characteristics. It is used to create three variables for the final dataset, namely the total number of words in the section and two binary variables indicating the occurrence of the words "renewable" and "sustainable" in the text. The TripAdvisor website uses customer ratings to create local rankings, which are displayed on each accommodation's page in the format "#X out of Y hotels in area". The raw variables *Ranking in area* and *Total in area* are used to create a score between 0 (included) and 1 (excluded) indicating the relative rating of a listing compared to other listings in the area. This relative score is included in the final dataset, the initial variables are discarded. In the above section, listings are further classified as *Hotel*, *B&B/Inn* or *Specialty Lodging*. This categorical variable is encoded into two binary variables in the final dataset. The variables *Specialty Lodging* and *B&B/Inn* are assigned the value 1 if they apply to a listing and 0 if not. This leaves the accommodation type *Hotel* as the base category, which is not one-hot encoded to avoid perfect multicollinearity.

In a listing's review section, TripAdvisor publishes summary data about the distribution of its ratings and lets users select comments in their languages of interest. The language subsection further shows up to four common review languages for the listing and the number of reviews written in each language. From this, nine raw variables can be retrieved: The total number of comments, the most prevalent review languages and the associated number of reviews in each language. The data is used to create two variables for the final dataset, the proportion of local language reviews and the proportion of English language reviews. For countries with multiple official languages, the sum of all reviews in the official languages is divided by the total number of reviews to calculate the proportion of local language reviews.

For countries with a single official language, the number of reviews in the local language is divided by the total number of reviews. The proportion of English language reviews follows the same logic.

An additional way for TripAdvisor page owners to inform potential customers about their accommodation is through the selection of available property amenities and room features. To the user, only facilities and features available at the accommodation are visible in the listing. Page owners can select from over 340 amenities and over 300 room features. From a small mixed sample of accommodations with and without the *GreenLeader* award, 34 amenities and 20 room features that differed most strongly in their prevalence between the two groups were selected. During the scraping of each listing, the two abovementioned sections are scanned for the selected features and amenities. From this, 54 one-hot encoded binary variables are created, with value 1 if the feature or amenity is available at the location and 0 if not. The same principle is used to control for the availability of seven different room types. The total number of available property amenities and room features is further included in the final dataset through variables recording the length of each section, irrespective of the exact facilities available.

Location on website (supplementary information I)	Raw Variable	Raw variable type, possible values	Transformation	Final variable: Type, Range	Variable in final dataset	Var #
**About Section**						
A1	Average Rating	Float (0-5)	/	Float (0-5)	Average Rating	1
A2	Location Rating	Float (0-5), steps of 0.5	/	Float (0-5), steps of 0.5	Location Rating	2
A3	Cleanliness Rating	Float (0-5), steps of 0.5	/	Float (0-5), steps of 0.5	Cleanliness Rating	3
A4	Service Rating	Float (0-5), steps of 0.5	/	Float (0-5), steps of 0.5	Service Rating	4
A5	Value Rating	Float (0-5), steps of 0.5	/	Float (0-5), steps of 0.5	Value Rating	5
A6	Traveler's Choice	Categorical (3 cat.): None, TC, 2020 TC Best	Create Binary variable where 0 if None, else 1	Binary, (0 / 1)	Traveler's Choice Award	6
A7	GreenLeader	Categorical (6 cat.): None, Green, Bronze, Silver, Gold, Platinum	Create Binary variable where 0 if None, else 1	Binary, (0 / 1)	GreenLeader Award (**y variable**)	
A8	Description	String	Count words	Integer	Description Length	7
			Check if section contains substring	Binary, (0 / 1)	"Sustainable" in Description	8
				Binary, (0 / 1)	"Renewables" in Description	9
A9	Hotel Class	Float (0-5), steps of 0,5	/	Float (0-5), steps of 0.5	Hotel Class	10
A10	Languages Spoken	List of languages and count of languages not listed	Sum number of languages listed + X more	Integer	Languages Spoken	11
A11	Hotel Style	List of strings describing hotel style	Check if list contains substring	Binary, (0 / 1)	Hotel Style "Green"	12
				Binary, (0 / 1)	Hotel Style "Business"	13

Location on website (supplementary information II)	Raw Variable	Raw variable type, possible values	Transformation	Final variable: Type, Range	Variable in final dataset	Var #
A11	Hotel Style	List of strings describing hotel style	Check if list contains substring	Binary, (0 / 1)	Hotel Style "Modern"	14
				Binary, (0 / 1)	Hotel Style "Luxury"	15
**Basics Section**						
B1	Name	String	/	/	/	
B2	Ranking in area	Integer	1- (Rank in region/ total number of listings in region)	Float (0-1)	Area Comparison	16
B3	Total in area	Integer				
B4	Accommodation Type "Specialty"	String	Check if text contains substring	Binary, (0 / 1)	Specialty Lodging	17
	Accommodation Type "B&B"				Bed & Breakfast	18
B5	Price Range Low	Integer, Price in Euro	/	Integer	Price Range Lower End	19
B6	Price Range High	Integer, Price in Euro	/	Integer	Price Range Upper End	20
B7	Country	String	/	/	/	
B8	State	String	/	/	/	
B9	Number of Rooms	Integer	/	Integer	Number of Rooms	21
**Location Section**						
L1	Walker Score	Integer (0-100), Score calculated by TripAdvisor	/	Score (0-100)	Walker Score	22
L2	Restaurant Score	Int, Number of Restaurants close by	/	Integer	Restaurant Score	23

<table>
<tr><td>L3</td><td>Attractions Score</td><td>Int, Number of Attractions close by</td><td colspan="2">/</td><td>Integer</td><td>Attractions Score</td><td>24</td></tr>
<tr><th>Location on website (supplementary information II)</th><th>Raw Variable</th><th>Raw variable type, possible values</th><th colspan="2">Transformation</th><th>Final variable:
Type, Range</th><th>Variable in final dataset</th><th>Var #</th></tr>
<tr><td>R1</td><td>Total number of reviews</td><td>Integer</td><td colspan="2">/</td><td>Integer</td><td>Review Count</td><td>25</td></tr>
<tr><td>R2</td><td>Number of Q&A comments</td><td>Integer</td><td colspan="2">/</td><td>Integer</td><td>Q&A Count</td><td>26</td></tr>
<tr><td>R3</td><td>Number of Room tips</td><td>Integer</td><td colspan="2">/</td><td>Integer</td><td>Room Tip Count</td><td>27</td></tr>
<tr><td>R4</td><td>Number of excellent reviews</td><td>Integer</td><td colspan="2">/</td><td>Integer</td><td>Excellent Reviews</td><td>28</td></tr>
<tr><td>R5</td><td>Number of very good reviews</td><td>Integer</td><td colspan="2">/</td><td>Integer</td><td>Very Good Reviews</td><td>29</td></tr>
<tr><td>R6</td><td>Number of average reviews</td><td>Integer</td><td colspan="2">/</td><td>Integer</td><td>Average Reviews</td><td>30</td></tr>
<tr><td>R7</td><td>Number of poor reviews</td><td>Integer</td><td colspan="2">/</td><td>Integer</td><td>Poor Reviews</td><td>31</td></tr>
<tr><td>R8</td><td>Number of terrible reviews</td><td>Integer</td><td colspan="2">/</td><td>Integer</td><td>Terrible Reviews</td><td>32</td></tr>
<tr><td>R9</td><td>Count of reviews in all languages</td><td>Integer</td><td rowspan="8">Create dictionary of local languages, calculate proportions</td><td rowspan="4">Local reviews / total reviews</td><td rowspan="4">Float (0-1)</td><td rowspan="4">Proportion of Local Reviews</td><td rowspan="4">33</td></tr>
<tr><td>R10</td><td>Most common review language</td><td>String</td></tr>
<tr><td>R11</td><td>Count of reviews in most common</td><td>Integer</td></tr>
<tr><td>R12</td><td>2nd most common review language</td><td>String</td></tr>
<tr><td>R13</td><td>Count of reviews in 2nd most common</td><td>Integer</td><td rowspan="4">English reviews / total reviews</td><td rowspan="4">Float (0-1)</td><td rowspan="4">Proportion of English Reviews</td><td rowspan="4">34</td></tr>
<tr><td>R14</td><td>3rd most common review language</td><td>String</td></tr>
<tr><td>R15</td><td>Count of reviews in 3rd most common</td><td>Integer</td></tr>
<tr><td>(not pictured)</td><td>4th most common review language</td><td>String</td></tr>
</table>

(not pictured)	Count of reviews in 4th most common	Integer				
**Location on website (supplementary information II)**	**Raw Variable**	**Raw variable type, possible values**	**Transformation**	**Final variable: Type, Range**	**Variable in final dataset**	**Var #**
**Pictures and Videos Section**						
P1	Total number of photos	Integer	/	Integer	Total Photos	35
P2	‘Traveler’ label	String	/	/	/	
P3	Count of above label pictures	Integer	/	Integer	Traveler Photos	36
P4	‘Room & Suite’ label	String	/	/	/	
P5	Count of above label pictures	Integer	/	Integer	Room & Suite Photos	37
P6	‘Dining’ label	String	/	/	/	
P7	Count of above label pictures	Integer	/	Integer	Dining Photos	38
(not pictured)	‘Pool & Beach’ label	String	/	/	/	
(not pictured)	Count of above label pictures	Integer	/	Integer	Pool & Beach Photos	39
(not pictured)	‘Video’ label	String	/	/	/	
(not pictured)	Count of above label pictures	Integer	/	Integer	Traveler Videos	40
**Features Section**						
F1	Property amenities	String	Length of amenities section	Integer	Amenities Section Length	41
			Check if section contains string	Binary, (0 / 1)	Amenities “Bicycle”	42
					Amenities “High-Speed Wi-Fi”	43

**Location on website (supplementary information II)**	**Raw Variable**	**Raw variable type, possible values**	**Transformation**	**Final variable: Type, Range**	**Variable in final dataset**	**Var #**
					Amenities “Restaurant”	44
F1	Property amenities	String	Check if section contains string	Binary, (0 / 1)	Amenities “Paid Parking”	45
					Amenities “Free Parking”	46
					Amenities “Fitness”	47
					Amenities “Bar”	48
					Amenities “Children Entertainment”	49
					Amenities “Pets”	50
					Amenities “Sauna”	51
					Amenities “Hot Tub”	52
					Amenities “Pool”	53
					Amenities “Coffee Shop”	54
					Amenities “Special Diet”	55
					Amenities “Breakfast”	56
					Amenities “Breakfast Room”	57
					Amenities “Taxi service”	58
					Amenities “Airport shuttle”	59
					Amenities “Business center”	60

Location on website (supplementary information II)	Raw Variable	Raw variable type, possible values	Transformation	Final variable: Type, Range	Variable in final dataset	Var #
					Amenities “Conference center”	61
F1	Property amenities	String	Check if section contains string	Binary, (0 / 1)	Amenities “Banquet”	62
					Amenities “Meeting rooms”	63
					Amenities “Terrace”	64
					Amenities “Baggage Storage”	65
					Amenities “Concierge”	66
					Amenities “Currency Exchange”	67
					Amenities “Non-smoking”	68
					Amenities “24 Hours”	69
					Amenities “Special Check-In”	70
					Amenities “Dry Cleaning”	71
					Amenities “Laundry”	72
					Amenities “Facial Treatment”	73
					Amenities “Spa”	74
					Amenities “Massage”	75
F2	Room features	String	Length of room features section	Integer	Room Feature Section Length	76
				Binary, (0 / 1)	Room feature “Allergy Free”	77

			Check if section contains string		Room feature “Flatscreen TV”	78
**Location on website (supplementary information II)**	**Raw Variable**	**Raw variable type, possible values**	**Transformation**	**Final variable: Type, Range**	**Variable in final dataset**	**Var #**
F2	Room features	String	Check if section contains string	Binary, (0 / 1)	Room feature “Walk-in Shower”	79
					Room Feature “Housekeeping”	80
					Room feature “Coffee and Tea”	81
					Room feature “Soundproof”	82
					Room feature “Safe”	83
					Room feature “Laptop Safe”	84
					Room feature “Balcony”	85
					Room feature “Refrigerator”	86
					Room feature “Toiletries”	87
					Room feature “Hairdryer”	88
					Room feature “Fireplace”	89
					Room feature “Air Conditioning”	90
					Room feature “Minibar”	91
					Room feature “Microwave”	92
					Room feature “VIP room”	93
					Room feature “Room Service”	94

					Room feature “Wake Up Service”	95
**Location on website (supplementary information II)**	**Raw Variable**	**Raw variable type, possible values**	**Transformation**	**Final variable: Type, Range**	**Variable in final dataset**	**Var #**
F3	Room types	List of strings of available room types	Check if section contains string	Binary, (0 / 1)	Room type “City View”	96
					Room type “Non-smoking”	97
					Room type “Suites”	98
					Room type “Family Room”	99
					Room type “Ocean View”	100
					Room type “Pool View”	101
					Room type “Bridal”	102

Supplementary Information III: Summary statistics for removed incomplete observations

While there are no obvious differences between the two groups in terms of hotel class and average total rating, significant differences can be found for other variables. The proportion of excluded listings of the category *Bed and Breakfast* is close to 40% while the accommodation type makes up little more than 20% of the included observations. Similarly, the mean *number of rooms* of complete observations is roughly double the mean value of excluded listings. There are also significant differences in the distribution of the *GreenLeader* label. The proportion of sustainable listings is roughly 4 times higher in the group of included observations than in the excluded group. The most significant differences, however, are found in variables related to user interaction. The mean number of user-uploaded pictures differs by a factor of four in favor of the included observations. The mean number of reviews differs by a factor of five in the same direction. Lastly, the single largest difference is found for listings' *Question & Answer* section, where included listings show more than eight times the amount of user interaction excluded listings show. In summary, the final subsample of 65,515 accommodations used for model training overestimates accommodation size and the closely related user interaction variables when compared to the initial sample. Hence, there are undeniable differences between the final sample and the full population that will have to be considered before any generalization can be made.

	Hotel Class		Average Rating		Price Range Lower End		Price Range Upper End		Bed & Breakfast		Specialty Lodging		Number of Rooms	
	included	removed	included	removed	included	removed	included	removed	included	removed	included	removed	included	removed
**Count**	65515	44748	65515	121045	65515	121565	65515	121565	65515	150291	65515	150291	65515	94278
**Mean**	3.10	3.04	4.06	4.17	76.59	72.62	162.97	132.54	0.22	0.38	0.12	0.27	55.07	24.96
**Std Dev**	0.85	0.81	0.59	0.82	49.22	47.05	157.68	133.20	0.42	0.48	0.32	0.44	75.18	50.99

	Total Photos		Dining Photos		Pool & Beach Photos		Room & Suite Photos		Traveler Photos		Traveler Videos		Review Count	
	included	removed	included	removed	included	removed	included	removed	included	removed	included	removed	included	removed
**Count**	65515	150291	65515	150291	65515	150291	65515	150291	65515	150291	65515	150291	65515	150291
**Mean**	230.57	51.94	10.97	3.46	5.13	1.42	57.87	10.39	163.29	35.32	0.08	0.02	408.96	76.91
**Std Dev**	382.19	147.04	24.75	11.34	31.21	14.28	101.34	31.54	312.47	120.63	0.45	0.21	681.69	232.87

	Excellent Reviews		Very Good Reviews		Average Reviews		Poor Reviews		Terrible Reviews		Q & A Count		Room Tip Count	
	included	removed	included	removed	included	removed	included	removed	included	removed	included	removed	included	removed
**Count**	65515	150291	65515	150291	65515	150291	65515	150291	65515	150291	65515	150291	65515	150291
**Mean**	192.22	40.09	130.01	20.74	50.97	8.54	19.50	3.89	16.28	3.65	15.50	1.93	25.35	4.79
**Std Dev**	362.70	118.38	226.88	72.15	96.62	35.88	40.79	18.14	46.22	18.14	71.18	20.18	33.46	15.40

	Location Rating		Cleanliness Rating		Service Rating		Value Rating		Walker Score		Restaurant Score		Attractions Score	
	included	removed	included	removed	included	removed	included	removed	included	removed	included	removed	included	removed
**Count**	65515	97930	65515	97982	65515	109171	65515	97998	65515	77939	65515	77939	65515	77939
**Mean**	43.63	43.24	43.02	43.83	41.81	42.40	40.58	41.87	77.26	74.78	91.66	87.27	27.14	26.91
**Std Dev**	4.84	6.45	5.54	7.31	5.47	7.92	5.41	7.63	20.35	20.98	128.22	119.38	46.14	45.75

	Description Length		"Sustainable" in Description		"Renewables" in Description		Languages Spoken		Hotel Style "Green"		Hotel Style "Business"		Hotel Style "Modern"	
	included	removed	included	removed	included	removed	included	removed	included	removed	included	removed	included	removed
**Count**	65515	150291	65515	150291	65515	150291	65515	150291	65515	150291	65515	150291	65515	150291
**Mean**	116.70	59.99	0.00	0.00	0.00	0.00	2.83	1.44	0.03	0.00	0.10	0.03	0.05	0.01
**Std Dev**	74.93	76.64	0.04	0.03	0.01	0.02	1.78	1.52	0.16	0.06	0.30	0.17	0.23	0.09

	Hotel Style "Luxury"		Room Type "City View"		Room Type "Non-Smoking"		Room Type "Suites"		Room Type "Family"		Room Type "Ocean View"		Room Type "Pool View"	
	included	removed	included	removed	included	removed	included	removed	included	removed	included	removed	included	removed
**Count**	65515	150291	65515	150291	65515	150291	65515	150291	65515	150291	65515	150291	65515	150291
**Mean**	0.01	0.00	0.05	0.01	0.73	0.31	0.28	0.11	0.43	0.27	0.09	0.02	0.02	0.01
**Std Dev**	0.08	0.03	0.22	0.12	0.45	0.46	0.45	0.32	0.50	0.44	0.29	0.14	0.15	0.10

	Room Type "Bridal"		Amenities Section Length		Amenities "Bicycle"		Amenities "High-Speed Wi-Fi"		Amenities "Restaurant"		Amenities "Paid Parking"		Amenities "Free Parking"	
	included	removed	included	removed	included	removed	included	removed	included	removed	included	removed	included	removed
**Count**	65515	150291	65515	150291	65515	150291	65515	150291	65515	150291	65515	150291	65515	150291
**Mean**	0.07	0.03	247.75	123.35	0.18	0.09	0.69	0.41	0.40	0.20	0.15	0.02	0.35	0.34
**Std Dev**	0.26	0.18	192.70	157.75	0.39	0.28	0.46	0.49	0.49	0.40	0.35	0.15	0.48	0.47

	Amenities "Fitness"		Amenities "Bar"		Amenities "Children Ent."		Amenities "Pets"		Amenities "Sauna"		Amenities "Hot Tub"		Amenities "Pool"	
	included	removed	included	removed	included	removed	included	removed	included	removed	included	removed	included	removed
**Count**	65515	150291	65515	150291	65515	150291	65515	150291	65515	150291	65515	150291	65515	150291
**Mean**	0.15	0.04	0.49	0.21	0.27	0.27	0.32	0.22	0.14	0.05	0.11	0.05	0.25	0.14
**Std Dev**	0.35	0.20	0.50	0.41	0.45	0.44	0.47	0.41	0.35	0.22	0.31	0.21	0.43	0.35

	Amenities “Coffee Shop”		Amenities “Special Diet”		Amenities “Breakfast”		Amenities “Breakfast Room”		Amenities “Taxi Service”		Amenities “Airport Service”		Amenities “Business”	
	included	removed	included	removed	included	removed	included	removed	included	removed	included	removed	included	removed
**Count**	65515	150291	65515	150291	65515	150291	65515	150291	65515	150291	65515	150291	65515	150291
**Mean**	0.10	0.04	0.14	0.07	0.64	0.28	0.20	0.07	0.10	0.02	0.17	0.09	0.15	0.05
**Std Dev**	0.30	0.20	0.34	0.25	0.48	0.45	0.40	0.25	0.30	0.14	0.37	0.29	0.35	0.21

	Amenities “Conference”		Amenities “Banquet”		Amenities “Meeting Rooms”		Amenities “Terrace”		Amenities “Baggage Storage”		Amenities “Concierge”		Amenities “Currency Exch.”	
	included	removed	included	removed	included	removed	included	removed	included	removed	included	removed	included	removed
**Count**	65515	150291	65515	150291	65515	150291	65515	150291	65515	150291	65515	150291	65515	150291
**Mean**	0.15	0.05	0.16	0.06	0.27	0.11	0.18	0.10	0.48	0.16	0.27	0.06	0.08	0.01
**Std Dev**	0.36	0.21	0.36	0.23	0.44	0.31	0.39	0.30	0.50	0.36	0.44	0.24	0.27	0.12

	Amenities “Non-Smoking”		Amenities “24 Hours”		Amenities “Special Check-In”		Amenities “Dry Cleaning”		Amenities “Landry”		Amenities “Facial Treatment”		Amenities “Spa”	
	included	removed	included	removed	included	removed	included	removed	included	removed	included	removed	included	removed
**Count**	65515	150291	65515	150291	65515	150291	65515	150291	65515	150291	65515	150291	65515	150291
**Mean**	0.65	0.35	0.35	0.07	0.19	0.08	0.26	0.05	0.41	0.14	0.05	0.01	0.13	0.05
**Std Dev**	0.48	0.48	0.48	0.26	0.39	0.28	0.44	0.23	0.49	0.35	0.22	0.12	0.33	0.21

	Amenities “Massage”		Room Feature Section Length		Room Feature “Allergy Free”		Room Feature “Flatsceen TV”		Room Feature “Walk-In Shower”		Room Feature “Housekeeping”		Room Feature “Coffee & Tea”	
	included	removed	included	removed	included	removed	included	removed	included	removed	included	removed	included	removed
**Count**	65515	150291	65515	150291	65515	150291	65515	150291	65515	150291	65515	150291	65515	150291
**Mean**	0.12	0.04	124.30	74.02	0.07	0.03	0.60	0.21	0.05	0.03	0.34	0.14	0.11	0.07
**Std Dev**	0.32	0.21	102.37	100.57	0.25	0.17	0.49	0.41	0.21	0.16	0.47	0.35	0.31	0.26

	Room Feature "Soundproof"		Room Feature "Safe"		Room Feature "Laptop Safe"		Room Feature "Balcony"		Room Feature "Refrigerator"		Room Feature "Toiletries"		Room Feature "Hairdryer"	
	included	removed	included	removed	included	removed	included	removed	included	removed	included	removed	included	removed
**Count**	65515	150291	65515	150291	65515	150291	65515	150291	65515	150291	65515	150291	65515	150291
**Mean**	0.15	0.06	0.46	0.12	0.04	0.01	0.21	0.05	0.25	0.16	0.26	0.14	0.36	0.18
**Std Dev**	0.36	0.25	0.50	0.33	0.21	0.09	0.40	0.21	0.43	0.36	0.44	0.35	0.48	0.39

	Room Feature "Fireplace"		Room Feature "Air Conditioning"		Room Feature "Minibar"		Room Feature "Microwave"		Room Feature "VIP Room"		Room Feature "Room Service"		Room Feature "Wake Up Service"	
	included	removed	included	removed	included	removed	included	removed	included	removed	included	removed	included	removed
**Count**	65515	150291	65515	150291	65515	150291	65515	150291	65515	150291	65515	150291	65515	150291
**Mean**	0.04	0.02	0.48	0.21	0.22	0.07	0.07	0.06	0.04	0.01	0.26	0.10	0.18	0.06
**Std Dev**	0.18	0.16	0.50	0.41	0.42	0.25	0.26	0.24	0.19	0.10	0.44	0.30	0.38	0.23

	Traveler's Choice Award		Area Comparison		Proportion of English Reviews		Proportion of Local Reviews		GreenLeader Award	
	included	removed	included	removed	included	removed	included	removed	included	removed
**Count**	65515	150291	65515	110139	65515	150291	65515	150291	65515	150291
**Mean**	0.33	0.08	0.60	0.54	0.40	0.24	0.55	0.52	0.04	0.01
**Std Dev**	0.47	0.27	0.27	0.26	0.31	0.32	0.33	0.40	0.19	0.10

Supplementary Information IV: Outlier candidates and adjustments

Hair and colleagues (2019) emphasize that outliers should only be removed if they are truly not representative of the population. Removing outliers too quickly may lead to a loss of generalizability. In the case of this analysis, little prior knowledge is available to determine the representativeness of the included accommodations' TripAdvisor pages. The lack of domain knowledge becomes especially apparent in the bivariate perspective: Is it unusual for a listing to have a very short description but a large proportion of English language reviews? Does a small bed and breakfast with a great TripAdvisor walker score represent a relevant segment of the population? Not enough work on the relationships between different aspects of hotels and their online presence has been done to answer these questions down to the level of variable distributions. Distant but correctly measured observations may be statistically influential outliers but at this point it is not possible to determine whether they are truly unique observations or representing a relevant group within the population. Accordingly, only erroneous data entries will be corrected or removed at this point.

The majority of variables retrieved from a listing's TripAdvisor page are automatically calculated, such as the number of reviews or the number of pictures uploaded by users. Other variables can be selected by page owners from a limited range of options, such as hotel class (X out of 5 stars) or the available room features (e.g.: Balcony available/ not available). Neither of these categories can be erroneously entered by page owners. A third category of variables is directly created from information available on other websites. Data entry errors may have occurred on those sites but investigating all partner sites would exceed the scope of this analysis. The only numerical variable included in the analysis, which page owners can freely and therefore erroneously enter, is the number of rooms available at the accommodation. For the univariate detection of outliers for this variable, a distributional correction is performed and all observations further than three standard deviations from the mean are flagged (see below). For 81 of the potential outliers, the large number of rooms can be confirmed through pictures of the accommodation, user reviews and external websites. Among these are camping grounds, conference center hotels, holiday resorts and student accommodations serving as hotels in the semester break. Of the remaining nine observations, five could be corrected using information found on other websites and in different sections of the same listings. The other four observations, among which are the two listings with the largest stated number of rooms, are removed from the data.

Accommodation name	Country	Number of rooms	Findings	Adjustment
Center Parcs les Hauts de Bruyeres	France	746	Large specialty lodging	/
Parque Santiago	Spain	748	Large holiday resort	/
DCU Rooms	Ireland	750	Student accommodation	/
Siblu Villages	France	750	Large holiday resort	/
Arkwright Hall	England	756	Student accommodation	/
Paris Marriott Rive Gauche	France	757	Hotel and conference center	/
Eri Beach & Village	Greece	769	Large holiday resort	/
Pontins Southport Holiday Park	England	769	Large holiday resort	/
Ibis Paris CDG Airport	France	772	Large airport hotel	/
Hotel Jaime I	Spain	775	Large holiday resort	/
Gran Hotel Bali	Spain	776	Large holiday resort	/
Rodos Palace	Greece	777	Large holiday resort	/
Camping Capalonga	Italy	778	Large specialty lodging	/
Domaine les Tamaris et les Portes du Soleil	France	780	Large holiday resort	/
Sheraton Parco de' Medici	Italy	782	Large holiday resort	/
Sol Pelicanos Ocas	Spain	783	Large holiday resort	/
Strand Palace	England	785	Large city hotel	/
Pure CityStay	England	785	Student accommodation	/
Hilton Birmingham Metropole	England	794	Hotel and conference center	/
Sol Principe	Spain	799	Large holiday resort	/
Coghurst Hall Holiday Park	England	800	Large holiday resort	/
Barricata Holiday Village	Italy	800	Large holiday resort	/
The Tower Hotel	England	801	Large city hotel	/
CDSHotels Terrasini - Citta del Mare	Italy	803	Large holiday resort	/
Camping Tohapi Le Castellas	France	805	Large specialty lodging	/
Clarion Hotel The Hub	Norway	810	Hotel and conference center	/
AC Hotel by Marriott Bella Sky Copenhagen	Denmark	811	Hotel and conference center	/
Hotel Riu Oliva Beach Resort	Spain	814	Large holiday resort	/
The Student Hotel Vienna	Austria	818	Student accommodation	/
Don Juan Resort	Spain	819	Large holiday resort	/
Hotel Hilton London Gatwick Airport	England	821	Large airport hotel	/
Hotel Riu Chicalana	Spain	832	Large holiday resort	/

**Accommodation name**	**Country**	**Number of rooms**	**Findings**	**Adjustment**
Copthorne Tara Hotel London Kensington	England	833	Large city hotel	/
Camping Club Les Sablons	France	853	Large specialty lodging	/
Pierre & Vacances Village Port-Bourgenay	France	862	Large holiday resort	/
Camping Bella Terra	Spain	867	Large holiday resort	/
Villages Nature Paris	France	868	Large holiday resort	/
Madrid Marriott Auditorium Hotel & Conference Center	Spain	869	Hotel and conference center	/
Lalandia Billund	Denmark	870	Large holiday resort	/
VOI Tanka Village	Italy	870	Large holiday resort	/
My Space Barcelona	Spain	900	Self-catering apartments	/
Maison du Lierre	France	900	Bed and Breakfast	No other information available; delete observation
Camping La Brise	France	900	Large specialty lodging	/
Hard Rock Hotel London	England	900	Large city hotel	/
Camping Sandaya Cypsela Resort	Spain	900	Large specialty lodging	/
Holiday Inn London Kensington Forum	England	906	Large city hotel	/
Siblu Villages - La Carabasse	France	906	Large specialty lodging	/
Melia Castilla	Spain	911	Large city hotel	/
Messonghi Beach Resort	Greece	920	Large holiday resort	/
Club del Sole	Spain	950	Large specialty lodging	/
Pullman Paris Montparnasse Hotel	France	957	Large city hotel	/
Estival Park Resort	Spain	968	Large holiday resort	/
Hyatt Regency Paris Etoile	France	995	Large city hotel	/
Disney's Hotel Cheyenne	France	1000	Large holiday resort	/
Alpin Aktiv Hotel Konigsleiten	Austria	1000	Hotel	50 rooms according to hotel’s website; change variable
Isamar Holiday Village	Italy	1000	Large holiday resort	/
Ergife Palace Hotel	Italy	1000	Hotel and conference center	/
Spiaggia D'Oro Camping & Village	Italy	1000	Large specialty lodging	/
David Russel Apartments	Scotland	1000	Self-catering apartments	/
Disney's Hotel Santa Fe	Paris	1000	Large holiday resort	/
Sheraton Frankfurt Airport Hotel & Conference Center	Germany	1008	Hotel and conference center	/
Disney’s Sequoia Lodge	France	1011	Large holiday resort	/
Park Inn by Radisson Berlin Alexanderplatz	Germany	1012	Large city hotel	/
Camping Les Sables d'Or	France	1015	Large specialty lodging	/
Park Plaza Westminster Bridge London	England	1019	Large city hotel	/

Accommodation name	Country	Number of rooms	Findings	Adjustment
Club Mac All Inclusive Family Resort	Span	1024	Large holiday resort	/
Queen’s Elms	Northern Ireland	1032	Student accommodation	/
OYO Brentwood Hotel	Scotland	1116	Hotel	The TripAdvisor description states 116 rooms; change variable
Estrel Berlin	Germany	1125	Hotel and conference center	/
Lopesan Costa Meloneras Resort, Spa & Casino	Spain	1136	Large holiday resort	/
Hotel Bastille Speria	Paris	1138	Small hotel	No alternative information available; delete observation
Gothia Towers	Sweden	1200	Hotel and conference center	/
Giardinin di Altea	Italy	1200	Large holiday resort	/
Barcelo Punta Umbria Beach Resort	Spain	1200	Large holiday resort	/
CABINN Copenhagen Hotel	Denmark	1202	Large city hotel	/
BelleVue Club	Spain	1468	Large holiday resort	/
Opal 1 & 2	England	1534	Self-catering apartments	/
Camping le Vieux Port	France	1546	Large specialty lodging	/
HF Tuela Porto	Portugal	1597	Hotel	194 rooms according to agoda.com; change variable
Butlin's Skegness Resort	England	1604	Large holiday resort	/
Sealands Caravan Park	England	1800	Large specialty lodging	/
Pollock Halls – Edinburgh First	Scotland	1800	Student accommodation	/
Stay QM	England	2000	Student accommodation	/
Hotel de Paris Monte-Carlo	Monaco	2019	Hotel	257 rooms according to Wikipedia article; change variable
Hotel Hiddenseer am Ozeaneum	Germany	2056	Hotel	26 rooms according to hrs.de; change variable
Union Lido Camping	Italy	3000	Large specialty lodging	/
The Thonet House	France	9800	Not a real place	Pictures do not match location, user revies claim scam; delete observation
Minerva Suites	Spain	9999	Not a real place	No pictures, no further details; delete observation

Supplementary Information V: Variable distributions

Three of the four classifiers used in this paper make distributional assumptions about the data. The random forest classifier is the only model in question still expected to perform well in the face of severe departures from normality. This alone warrants a closer inspection of the variable distributions. In addition, their review can aid in building domain knowledge for tourism-related platform data. In the following, the distributions of the 33 variables treated as continuous in the modelling stage are briefly discussed. Additionally, the application of power transformations and their ability to alter variable distributions are shown by use of an example variable. Please note that only variables treated as continuous are included. Binary variables are not pictured. For a further discussion of the threats to validity from the inclusion of binary variables please refer to Van Belle and Fisher (2004). The 33 variables in question fall into one of three categories: Ordinal variables following a roughly normal distribution; Count-type variables, which fall close to zero for most observations but reach very high values for a select few listings; Proportion or capped variables, which tend to have two distributional centers.

Most ordinal variables following a roughly normal distribution are ratings. These include the externally determined *hotel class* ranging from one to five stars as well as user-generated ratings for location, cleanliness, service, value and overall quality in the same range. Further inspection reveals that the respective distribution means are found towards the upper end of the evaluation range (3.1 for hotel class; all user-generated between 4.0 and 4.5). This phenomenon is consistently found in rating scores from other online platforms. Teubner and Glaser (2018) review the 'up or out' dynamic of rating scores for Airbnb ratings. They find that high rates of market exit for low reputation sellers attribute to a population of highly rated survivors. Additional research about the entry and exit of TripAdvisor listings would be required to confirm a similar dynamic, which exceeds the scope of this paper. Nonetheless, the leptokurtic and negatively skewed characteristics of the rating distributions are worth noting before moving ahead.

Count-type variables or those following similar distributional patterns dominate the continuous portion of the dataset. They include language counts, the length of descriptive sections, the number of pictures uploaded in different sections, the number of reviews by category and the number of rooms as well as average room prices. The above variable types show a highly significant positive skew with a wide range of realized values but very few observations in the upper portions of the distribution. The maximum number of *total reviews*, for example, is over 20,000 while close to 90% of listings have less than 1,000 reviews and

half of all listings have less than 180 reviews. The choice for variable transformations will be made with the help of parameterized variable transformation frameworks and the improvement of classifier performance as the final goal. In order to get a first understanding of the effect of power transformations applied to the continuous explanatory variables, example adjustments for the *total reviews* variable were created. The following figure compares the variable's original distribution with a square root transformation and a logarithmic transformation:

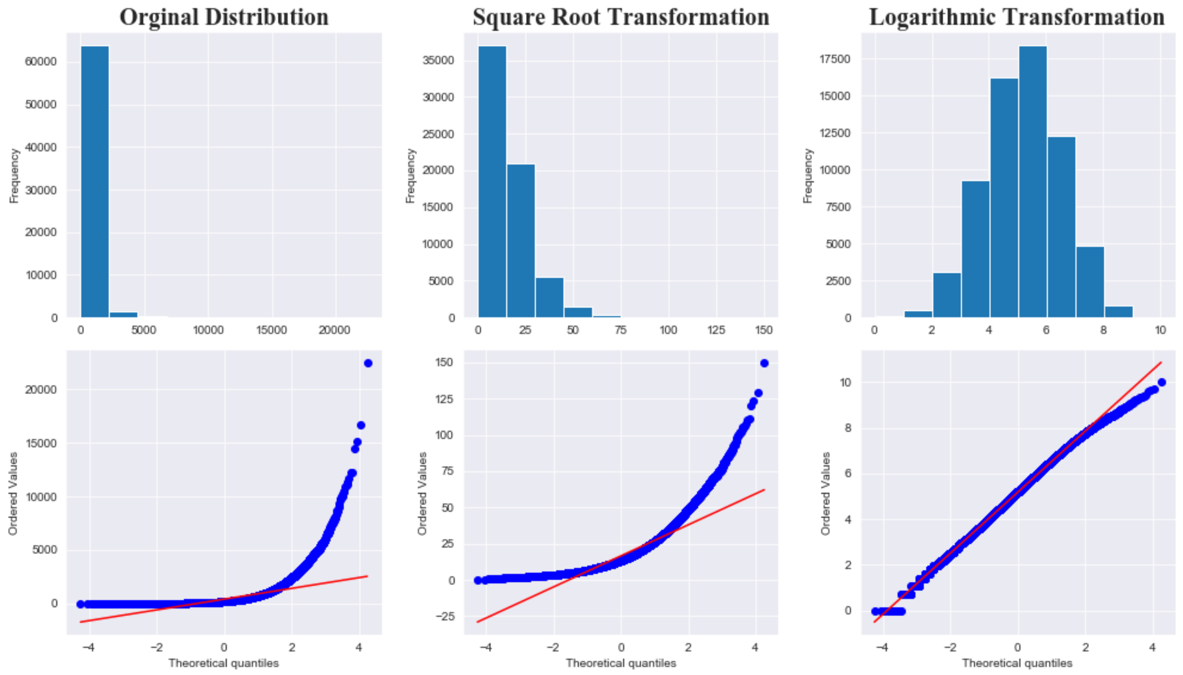

Figure 1: Transformation of the review count variable

The unaltered distribution to the left deviates most strongly from normality. The histogram on top shows that the vast majority of observations are found in the first bin, with the second and third bins barely visible and so few higher values that their respective bins are not visible at all. The probability plot in the bottom shows the difference between realized values in blue and the best-fit normal distribution in red. For the original distribution, significant differences from a theoretical normal distribution are found both at the minimum value of zero and for large values at the upper end of the distribution. By taking the square root of each value, the variable distribution can be transformed to be more Gaussian-like. The kurtosis is visibly smaller in the second histogram and the best-fit normal distribution more closely fits the data. However, there are still significant departures from normality. The logarithmic transformation shows yet stronger qualities for the transformation towards a normal distribution. Even though the transformation overshoots the mark and causes the variable distribution to be platykurtic and negatively skewed, the distribution of the transformed variable is most closely aligned with the best-fit normal distribution. While clearly performing best for the transformation of the *review*

count variable, the logarithmic transformation will not be the best fit for all variables. The Box-Cox and Yeo-Johnson transformations automate the above comparison and evaluate additional techniques.

The last category of distributions with similar characteristics entails proportions and capped variables. For the *proportion of reviews in the local language(s)* and *proportion of English-speaking reviews* variables, two distributional centers are found. The proportion of local reviews peaks for values close to zero and values close to one. Similarly, reviews in the English language make up a small portion of total reviews for many listings but close to 100% of reviews for another large group. The number of *room tips* follows a similar distribution because of the way TripAdvisor displays the variable. For values up to 100, the true number of tips is displayed. For listings with more tips, the displayed number is capped and set equal to 100 on the listing's page. As a result, the variable distribution has a large peak for very low numbers and a secondary peak at the maximum possible value. In the same way as the abovementioned variables, any transformation of proportion and capped variables will be evaluated on the improvement of model prediction capabilities.

Graphical comparison of univariate distributions

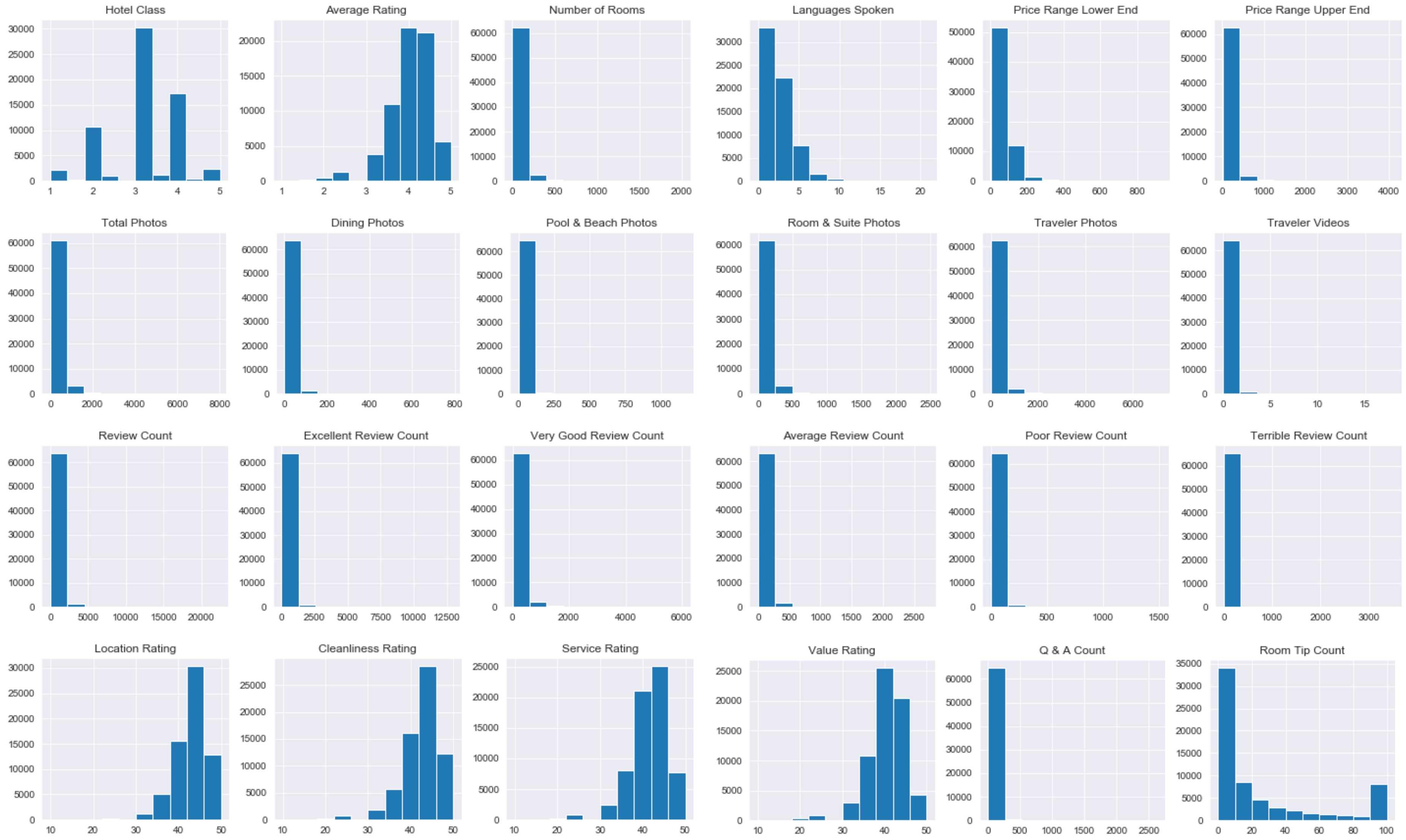

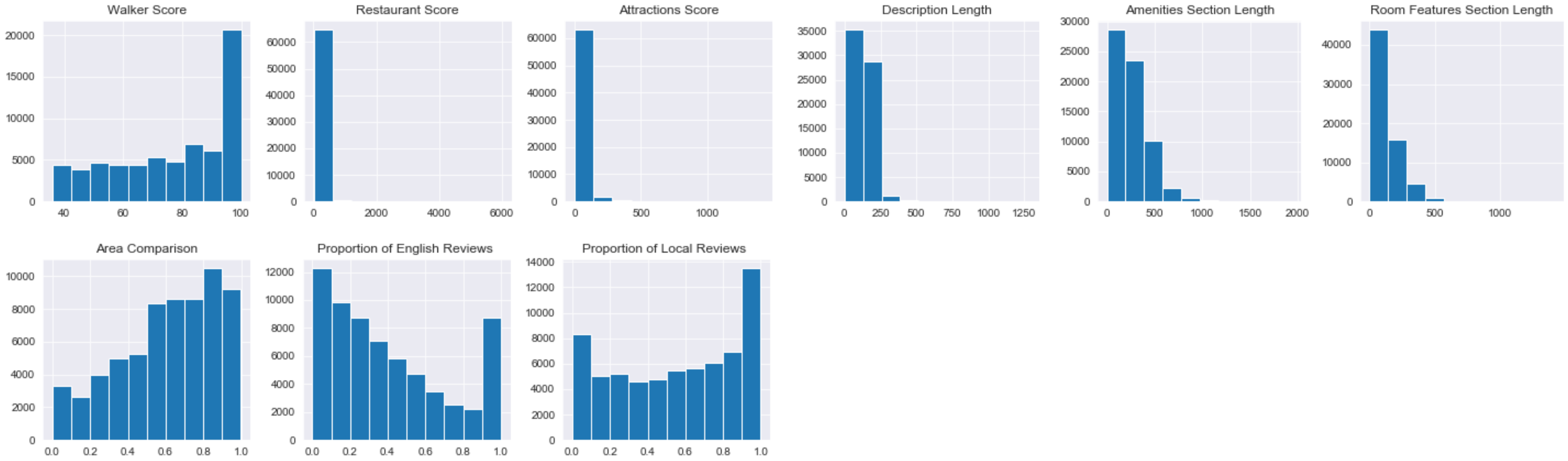
Walker Score
0
5000
10000
15000
20000
40
60
80
100
Restaurant Score
0
10000
20000
30000
40000
50000
60000
0
2000
4000
6000
Attractions Score
0
10000
20000
30000
40000
50000
60000
0
500
1000
Description Length
0
5000
10000
15000
20000
25000
30000
35000
0
250
500
750
1000
1250
Amenities Section Length
0
5000
10000
15000
20000
25000
30000
0
500
1000
1500
2000
Room Features Section Length
0
10000
20000
30000
40000
0
500
1000
Area Comparison
0
2000
4000
6000
8000
10000
0.0
0.2
0.4
0.6
0.8
1.0
Proportion of English Reviews
0
2000
4000
6000
8000
10000
12000
0.0
0.2
0.4
0.6
0.8
1.0
Proportion of Local Reviews
0
2000
4000
6000
8000
10000
12000
14000
0.0
0.2
0.4
0.6
0.8
1.0

Correlation between variables

GreenLeader Badge and numeric variables:

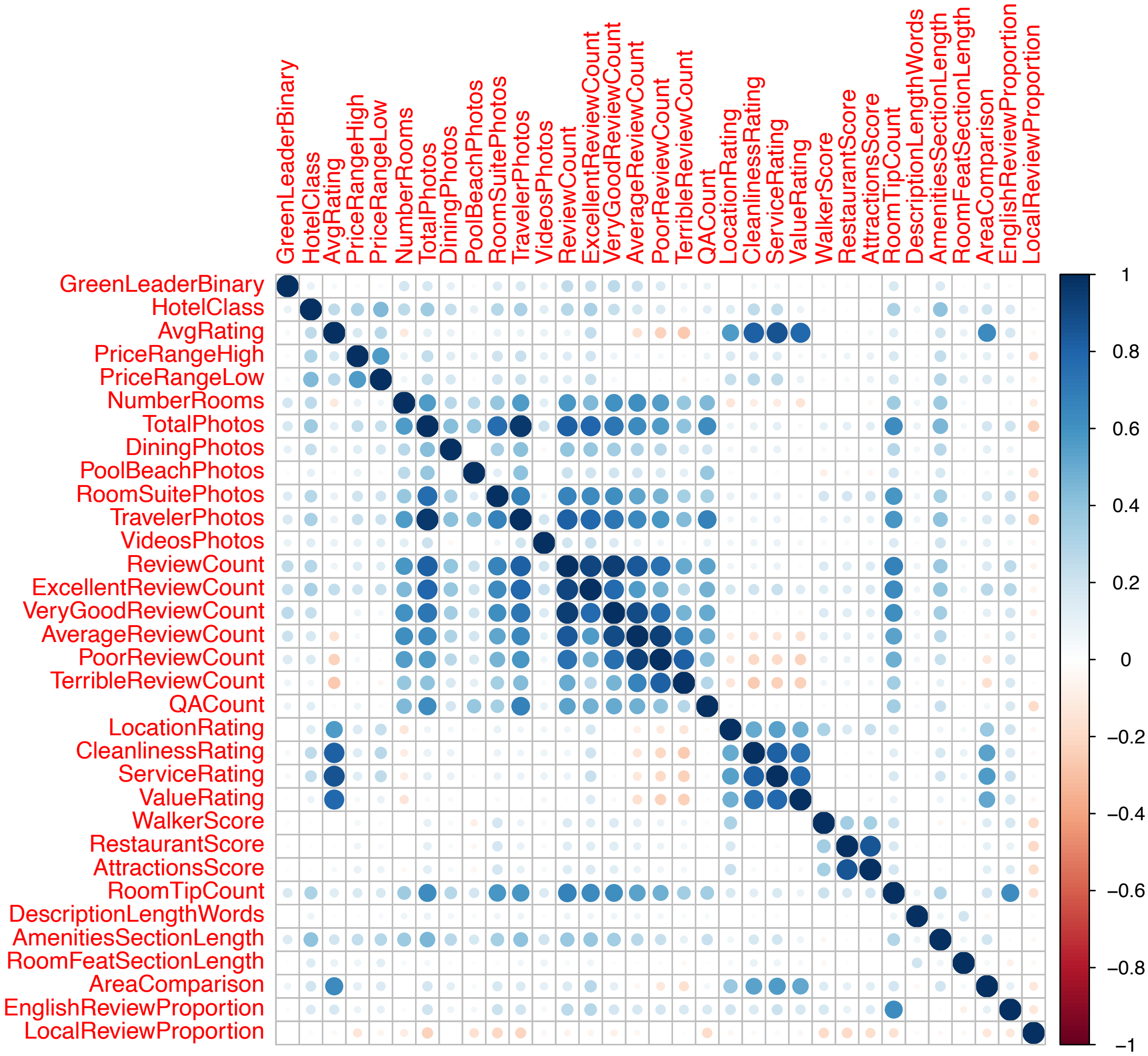

GreenLeader Badge and a set of binary variables:

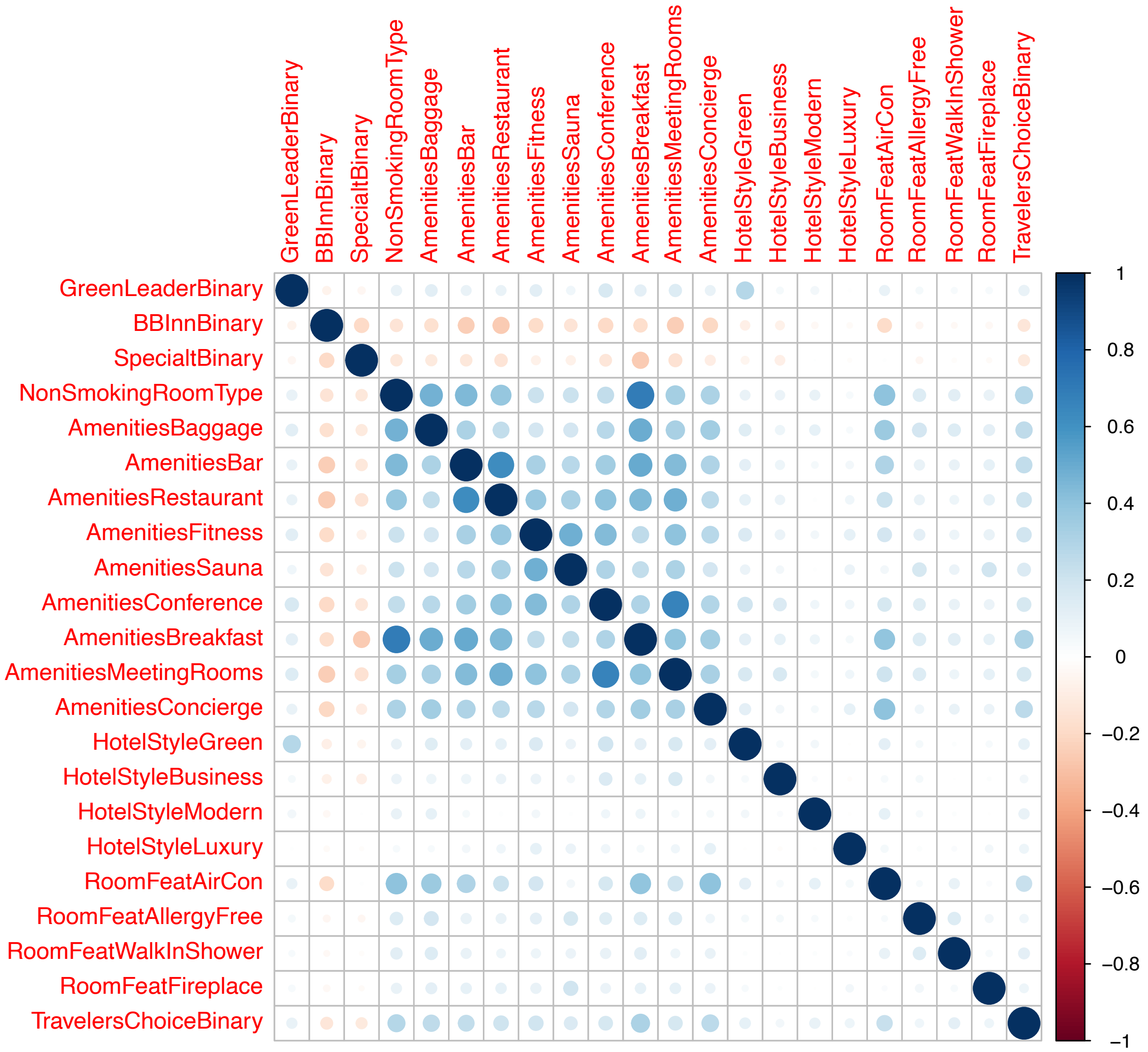

Supplementary Information VI: Description of pre-processing techniques used in grid-search

Dimensionality Reduction

Principal Component Analysis (PCA) is a dimensionality-reduction technique that can be used both in the exploratory stage of data-based research and as a preparatory step in the modelling process. The underlying idea is that a small number of well-chosen dimensions may be able to carry a large portion of the available information while simultaneously avoiding issues caused by high dimensionality of the original data. PCA aims to do this by finding the directions along which observations show the highest variance. An alternative interpretation is that the first principal component (PC) is the line which fits as close as possible to the available data.

When principal component analysis is applied as a preparatory step for the creation of a model, a small number of dimensions is not only assumed to explain a large proportion of the variation in the data but to also have a strong relationship with the response variable. Instead of using the original input variables as predictors, the principal components take their place. If the abovementioned assumptions hold, this will lead to better generalization results. This is possible because most of the information will be contained while the reduced number of estimated coefficients can aid in the mitigation of overfitting. The transformation is most effective for increasing a classifier's prediction capabilities when the first few principal components capture most of the variation in the data (James et al., 2013). When PCA is used as an exploratory tool, insights can be gained from the analysis of correlations. The correlation between a principal component and a given variable can be interpreted as shared information. By design, the sum of squared correlation coefficients between a given variable and all PCs is equal to one. Accordingly, the correlations can give an overview of variables relative influence on each dimension and inform which variables affect the most important dimensions (Abdi & Williams, 2010).

Sampling Methods

In order to prevent classifiers from learning that minority class observations are scarce and not important, the analysis of an imbalanced dataset requires extra steps. A range of methods is available both for the transformation of the dataset and for the modification of the classifiers. *Sampling* methods are part of the first group. They focus on changing the composition of the training data, aiming for a balance of class frequencies. The name *sampling* stems from the re-sampling of an already existing dataset. Sampling methods ignore possible causes of imbalance in the domain specific to the problem. Instead, the issue of relative class imbalance is solved

in a widely applicable manner. This can be of great benefit to the later analysis as it allows for the application of standard, unmodified machine learning algorithms to imbalanced domains (He & Ma, 2013). It is important to note that sampling methods are only applied to the training data. In order to get a fair understanding of classifier performance, class proportions in the holdout data remain representative of the full sample. In the following, the random under- and oversampling and synthetic minority oversampling techniques will be introduced.

Random data sampling or *naïve sampling* techniques offer the easiest implementation and execution of the discussed methods. No assumptions about the data are made and the fast execution is a desirable feat for large datasets. The methods work through the deletion or duplication of existing observations. *Random undersampling* balances class frequencies through the deletion of majority class observations. As no additional examples are added, the method is more suitable for relative class imbalance with a sufficient absolute number of minority class observations. Since observations are selected randomly for deletion, no mechanism to preserve examples especially rich in information is in place. This can lead to the deletion of large portions of useful data. The *Random oversampling* technique on the other hand selects and duplicates examples from the minority class and adds the resulting observations to the training set. The selection is performed randomly and with replacement so that original observations may be duplicated multiple times. Accordingly, random oversampling is effective when multiple realizations with the same properties can affect the classifier. This is the case for models using iterative optimization and for classifiers using data splits, such as decision trees (Brownlee, 2020). With strong imbalances, random oversampling can cause a model to overfit minority class observations. This risk should be kept in mind to avoid the loss of generalization ability.

While random oversampling manages to balance class frequencies without loss of data, it provides no additional information. The *Synthetic Minority Oversampling Technique (SMOTE)* improves upon the simple duplication of observations by creating new, unique observations on the basis of existing data. This is done by first randomly selecting an example from the minority class and determining its k nearest neighbours, where k is a hyperparameter and typically small. In the next step, a single observation is randomly selected from the set of neighbours and a line between the original observation and the selected neighbour is drawn in feature space. In the final step, a new, synthetic observation is sampled at a point along the line (Brownlee, 2020). While advantageous in many situations, adding a synthetic observation to the dataset also brings about risks. Since new examples are created without consideration of the majority class, SMOTE may create ambiguous observations in areas where classes have a

strong overlap. Finally, it is worth considering the application of multiple sampling techniques to the same set of data. Employing both an under – and an oversampling method has been shown to achieve better results than focusing on one technique (Chawla et al., 2002).

Transformations

The difficulty of a modelling problem may be increased by differences in scale and distribution of the included variables. A large proportion of popular algorithms performs better when all numerical inputs are scaled to the same range. Algorithms affected by this include those using a weighted sum of the input to predict outputs, such as linear regression, logistic regression, and artificial neural networks. The second group of algorithms requiring scaled data for good performance works on the basis of distance measures. This group includes the *k-nearest neighbors* algorithm and *support vector machines*. However, not all approaches are affected. Tree-based methods such as decision trees and random forests do not require scaled variables (Brownlee, 2016). In the following, data transformations through normalization, standardization and robust standardization will be introduced. The section then moves to generalized power transformations, which aim to make variable distributions more Gaussian.

Normalization is the most straightforward method of scaling input data. Through a simple calculation, all values are scaled to a chosen range. The calculation requires variables' maximum and minimum values. The final scaled variables are calculated as $y = (x - min)/(max - min)$. Maximum and minimum values can be based on the available sample but ideally also consider domain knowledge. The reason for this becomes apparent when a normalization procedure fit on sample data is applied to a variable outside of the sample range. In this case the resulting y will not fall into the intended output range. It is therefore important to review the employed values or guarantee for their validity by limiting input values (Brownlee, 2016). *Standardization* or center scaling may be more robust in the face of new data than normalization. The technique works by subtracting the variable mean (centering) and dividing by the standard deviation (scaling). Formally, $z = (x - \mu)/\sigma$, where mean and standard deviation are calculated based on the training data as $\mu = \frac{1}{N}\sum_{i=1}^{N} x_i$ and $\sigma = \sqrt{\frac{1}{N}\sum_{i=1}^{N}(x_i - \mu)^2}$. Standardization assumes that variable distributions can be fit to a Gaussian distribution. This includes having a well-behaved mean and standard deviation. It is possible to standardize variables not fulfilling these assumptions, but the achieved results may not be reliable. Outliers also pose a risk to standardization as they tend to skew either mean, standard deviation or both. A solution to this is *robust scaling*, which relies on *median* and *interquartile*

range (IQR) instead of mean and standard deviation. Formally, $y = (x - median)/IQR$, where the median is the middle value of the sample distribution. The interquartile range describes the difference between the 75th and the 25th percentile of the distribution. Similar to standardization, robust scaling will output a distribution with zero mean and median. Unlike standardization however, outliers will neither skew the output nor lose their relative relationship to other datapoints (Brownlee, 2016). Which scaler performs best in terms of classifier performance will not be clear from the beginning. Brownlee (2016) suggests exploring the effect of different scaling techniques on the final model's prediction capabilities and deciding accordingly.

The abovementioned techniques aim at scaling data so that variable distributions fall into the same range and fulfil simple model assumptions. Some statistical learning techniques, such as logistic regression and the Naïve Bayes classifier, extend assumptions to include a Gaussian distribution of input variables. A second group of models does not explicitly require normally distributed data but tends to perform better in its presence (Brownlee, 2016). It is therefore worthwhile to review *power transformations*, which employ logarithmic and exponential transformations to remove variable skew and stabilize variance. Such transformations can be achieved via the direct application of the logarithm, square root or others to a given variable. While quickly realized, this may not always lead to the use of the optimal power transform for each variable. Instead, generalized approaches, which find the optimal transformation through parameter tuning, have been developed. This paper employs two approaches for the use of parameters in making variable distributions more Gaussian, the *Box-Cox transformation* and the *Yeo-Johnson transformation*.

The Box-Cox transformation requires strictly positive input. It is formally defined as

$$y_i^{(\lambda)} = \begin{cases} \dfrac{(y_i)^\lambda - 1}{\lambda} & \text{for } \lambda \neq 0 \\ \ln(y_i) & \text{for } \lambda = 0 \end{cases}$$

where the parameter λ is estimated according to the training data (Box & Cox, 1964). Depending on the value used, a reciprocal transform, log transform, square root transform, a range of combinations or no transformation at all can be employed. Optimal values found in the training data are saved and later applied to new data. To guarantee strictly positive input data, a preprocessing step scaling all variables to the range between one and two will be used beforehand. The Yeo-Johnson transformation follows a similar principle but does not require pre-processing as it also allows for zero and negative input values.

It is formally given by:

$$y_i^{(\lambda)} \begin{cases} ((y_i+1)^{\lambda}-1)/\lambda & \text{if } \lambda \neq 0, y \geq 0 \\ \log(y_i+1) & \text{if } \lambda = 0, y \geq 0 \\ -[(-y_i+1)^{(2-\lambda)}-1]/(2-\lambda) & \text{if } \lambda \neq 2, y < 0 \\ -\log(-y_i+1) & \text{if } \lambda = 2, y < 0 \end{cases}$$

where the optimal parameter λ is once again estimated using the training data (Yeo & Johnson, 2000). As the improvement of prediction quality is the core goal of this paper, all data transformation and their combinations will be evaluated by their ability to improve final model output.

Supplementary Information VII: Description of classifiers used in grid-search

Logistic Regression

The first considered statistical modelling technique is Logistic Regression. It is a supervised learning technique, meaning that group membership is known in the training data. Logistic regression has two complimentary objectives: Classification and explanation. The final evaluation and comparison to other techniques will be performed on the basis of classification performance.

A binary classification could be approached through a linear regression model if the response variable is coded to values 0 and 1 using the *dummy variable* approach. In the case of this paper, the categorical outcome *GreenLeader* would be coded to the numerical value 1 and *not GreenLeader* would be coded to 0.

Modelling the binary response with least squares regression like this is not without merit. It provides a simple solution to the problem and $X\hat{\beta}$ is indeed an estimate of the probability that the regressand takes value 1 (James et al., 2013). However, this approach violates two assumptions of linear regression that cannot be remedied through transformation of the variables. Firstly, the error term of the discrete variable does not follow the normal distribution, rendering statistical testing based on the assumption of normality invalid. Secondly, the variance of a Bernoulli random variable is a function of of $X_1, \ldots, X_k$, and therefore heteroscedastic by definition (Hair et al., 2019). To avoid these shortcomings, an alternate model needs to be able to accommodate the binomial distribution and heteroscedasticity. In addition, the function used in modelling the probability should return outputs between 0 and 1 for all input values. Logistic regression fulfils these requirements.

In its standard form, the underlying *logistic function* is expressed as:

$$f(x) = \frac{1}{1 + e^{-x}} = \frac{e^{x}}{1 + e^{x}}$$

Filling in the linear regression model with an example model using a single regressor, the full formula is given by:

$$p(X) = \frac{e^{\beta_0 + \beta_1 X}}{1 + e^{\beta_0 + \beta_1 X}}$$

The calculation of the probability with a range of input values makes clear that it will be between 0 and 1 for every input level. The function will return an S-shaped curve, which gets close to but never becomes 0. The output value will approach 1 with increased input values but never fully reach it (James et al., 2013).

Linear Discriminant Analysis

The second modelling technique considered for the classification of observations is linear discriminant analysis (LDA). Like logistic regression, LDA is both a supervised learning technique and a classification method. Accordingly, a model is trained with labelled datapoints in order to later correctly assign unlabeled observations to the available classes.

While logistic regression directly models the conditional distribution of the response class given the explanatory variables, LDA follows a less direct approach. The technique makes use of differences in the distribution of the predictor variables between the response groups. This follows the intuition that the distribution of X given Y can offer information about the distribution of Y given X (James et al., 2013). The relevant theorem by Bayes states:

$$\Pr(Y = k|X = x) = \frac{\pi_k f_k(x)}{\sum_{l=1}^{K} \pi_l f_l(x)}$$

LDA assumes that $X = (X_1, X_2, \dots, X_p)$ is drawn from a *multivariate normal* or *Gaussian* distribution. In addition, LDA assumes a common covariance matrix $\boldsymbol{\Sigma_k} = \boldsymbol{\Sigma}\ \forall\ k$ and a class-specific mean vectors. Formally, the multivariate Gaussian density is denoted as:

$$f(x) = \frac{1}{(2\pi)^{\frac{p}{2}}|\boldsymbol{\Sigma}|^{\frac{1}{2}}} \exp\left(-\frac{1}{2}(x-\mu)^T\boldsymbol{\Sigma}^{-1}(x-\mu)\right)$$

LDA relies on the estimation of the parameters in the above formula from the available sample data. In particular, the method estimates the prior probability of a chosen observation coming from the kth class as the proportion of observations belonging to class k in the training set. After plugging all available information into Bayes' Theorem and rearranging, it can be shown that the classifier assigns the observation x to the class for which

$$\delta_k(x) = x^T\boldsymbol{\Sigma}^{-1}\mu_k - \frac{1}{2}\mu_k^T\boldsymbol{\Sigma}^{-1}\mu_k + log\pi_k$$

returns the largest value. As suggested by the term *linear* discriminant analysis, the classification rule depends on x through the linear combination of the observation's elements.

Quadratic Discriminant Analysis

The third classifier considered is quadratic discriminant analysis (QDA). Like LDA, QDA is based on the assumption that the observations in a given class are drawn from the Gaussian distribution. QDA also makes use of Bayes' Theorem, again resembling the LDA procedure. Unlike linear discriminant analysis however, quadratic discriminant analysis does not assume a common covariance matrix for observations of all classes and instead allows for different

covariance matrices for each class. Formally, a given observation will be of the form $X \sim N(\mu_k, \Sigma_k)$, where both the mean and the covariance matrix are class-dependent (James et al., 2013).

(James et al., 2013). This change of assumptions has a significant effect on the complexity of the classifier's decision rule. assuming class-dependent covariance matrices, an observation is assigned to the class for which the following is largest (Friedman et al., 2001):

$$\delta_k(x) = -\frac{1}{2}(x-\mu_k)^T \mathbf{\Sigma}_k^{-1}(x-\mu_k) - \frac{1}{2} log|\mathbf{\Sigma}_k| + log\pi_k$$

Random Forest Classifier

The final method included in the analysis is the random forest classifier. It is an ensemble method and an algorithmic modelling approach. As such, the method fundamentally differs from the methods introduced earlier. Tree-based methods work through the segmentation of the predictor space. This segmentation is achieved through a stepwise process based on previously determined criteria. In the case of classification trees, the optimal next cutting point is determined based on measures of impurity.

Low variance is a desirable attribute for an estimation procedure because similar results can be achieved even when using distinct data sets. Decision trees alone are not able to perform well in this regard but can be improved upon by *bootstrap aggregation* or *bagging*. To understand the advantages of the additional effort, it is useful to recall that, given n independent observations with variance σ^2, the mean of these observations has variance σ^2/n. Accordingly, a reduction in variance can be achieved through averaging over observations. In the regression context, this translates to using multiple training sets to build separate models and ultimately averaging over them. In classification, the same effect can be achieved by letting multiple models participate in a majority vote to determine the final class estimate. The advantages of this process can be likened to the *wisdom of crowds*-concept, supposing that collective knowledge exceeds individual knowledge given sufficient diversity of the crowd (James et al., 2013). In order to perform this method using a single training set, repeated subsamples comprised of different combinations of the original observations are taken. This process is called *bootstrapping*. Finally, the combination of bootstrapped sampling and moving ahead with the average or majority vote prediction is known as bootstrap aggregation or bagging.

Any correlation between decision trees limits the effect of bootstrap aggregation. The principle of the random forest method is to improve the reduction of variance from bagging by

decorrelating trees (Hastie et al., 2009). This decorrelation is achieved through a small tweak in the growing stage of the decision trees. Before each split, a subset m of the p predictors is chosen at random. This split is chosen so that the majority of predictors will not be considered in each step, usually $m = \sqrt{p}$. While simple decision trees have the advantage of easily interpretable diagrams, this benefit is lost through bagging. The random forest method's primary purpose in this paper is the correct classification of new observations. Accordingly, trading interpretability for prediction accuracy is an acceptable step. The full random forest for classification algorithm is defined as follows (Hastie et al., 2009):

Random Forest for Classification Algorithm

1. For $b = 1$ to B trees:
   (a) Draw a Bootstrap sample $\boldsymbol{Z}^*$ of size N from the training data.
   (b) Grow a random-forest tree T_b to the bootstrapped data by recursively repeating for each terminal node until minimum node size n_{min} is reached.
      i. Select m variables at random from the p variables.
      ii. Pick the best variable/split point among the m.
      iii. Split the node into two daughter nodes.
2. Output the ensemble of trees $\{T_b\}_1^B$.
3. To make a new class prediction at point x,
   (a) let $\hat{C}_b(x)$ be the class prediction of the bth random-forest tree and
   (b) let $\hat{C}_{rf}^B(x) = majority\ vote\ \{\hat{C}_b(x)\}_1^B$ be the estimated class.

Supplementary Information VIII: Comparison of variable distributions between GreenLeaders and non-GreenLeaders in final sample

	Hotel Class		Average Rating		Price Range Lower End		Price Range Upper End		Bed & Breakfast		Specialty Lodging		Number of Rooms	
	GL	Non-GL	GL	Non-GL	GL	GL	GL	Non-GL	GL	Non-GL	GL	Non-GL	GL	Non-GL
**Mean**	3.52	3.09	4.18	4.05	85.1	76.3	182	162	0.09	0.23	0.04	0.12	127	52.4
**Std. Dev.**	0.85	0.85	0.43	0.59	47.8	49.2	149	157	0.29	0.42	0.19	0.33	121	71.5
**Median**	4	3	4	4	73	66	146	125	0	0	0	0	98	29.0
**Min**	1	1	2.5	1	15	1	35	13	0	0	0	0	1	1
**Max**	5	5	5	5	433	931	4084	4106	1	1	1200	2000	1200	2000

	Total Photos		Dining Photos		Pool & Beach Photos		Room & Suite Photos		Traveler Photos		Traveler Videos		Review Count	
	GL	Non-GL	GL	Non-GL	GL	GL	GL	Non-GL	GL	Non-GL	GL	Non-GL	GL	Non-GL
**Mean**	579	217	24.5	10.5	8.37	5	133	55.0	435	153	0.30	0.07	1292	376
**Std. Dev.**	720	357	43.4	23.6	55.2	29.9	179	96.0	637	288	0.82	0.43	1393	615
**Median**	327	91	9	3	0	0	61	19	218	62	0	0	877	171
**Min**	6	0	0	0	0	0	0	0	0	0	0	0	1	0
**Max**	7890	6356	477	785	1172	1094	2476	1926	7198	5974	16	18	22499	16649

	Excellent Reviews		Very Good Reviews		Average Reviews		Poor Reviews		Terrible Reviews		Q & A Count		Room Tip Count	
	GL	Non-GL	GL	Non-GL	GL	GL	GL	Non-GL	GL	Non-GL	GL	Non-GL	GL	Non-GL
**Mean**	604	177	444	118	158	46.9	52.3	18.3	33.2	15.6	44.9	14.4	53.4	24.3
**Std. Dev.**	779	327	469	203	190	88.7	76.5	38.3	54.5	45.8	144	66.6	39.7	32.7
**Median**	342	68	320	48	110	17	30	6	17	5	11	2	47	8
**Min**	1	0	0	0	0	0	0	0	0	0	0	0	0	0
**Max**	12836	10210	6023	5020	2720	2613	12352/0	1518	864	3506	2265	2625	100	100

	Location Rating		Cleanliness Rating		Service Rating		Value Rating		Walker Score		Restaurant Score		Attractions Score	
	GL	Non-GL	GL	Non-GL	GL	GL	GL	Non-GL	GL	Non-GL	GL	Non-GL	GL	Non-GL
**Mean**	43.8	43.6	44.3	43.0	43.1	41.8	41.0	40.6	80.3	77.2	105	91.2	30.8	27.0
**Std. Dev.**	4.34	4.86	3.60	5.60	3.82	5.51	3.83	5.46	20.6	20.3	120	129	44.8	46.2
**Median**	45	45	45	45	45	40	40	40	88	81	58	46	14	11
**Min**	25	10	30	10	30	10	25	10	36	36	5	2	0	0
**Max**	50	50	50	50	50	50	50	50	100	100	1935	6011	519	1420

	Description Length		"Sustainable" in Description		"Renewables" in Description		Languages Spoken		Hotel Style "Green"		Hotel Style "Business"		Hotel Style "Modern"	
	GL	Non-GL	GL	Non-GL	GL	GL	GL	Non-GL	GL	Non-GL	GL	Non-GL	GL	Non-GL
**Mean**	117	117	0.01	0.00	0.00	0.00	3.49	2.80	0.26	0.02	0.17	0.10	0.12	0.05
**Std. Dev.**	70.0	75.1	0.11	0.04	0.02	0.01	2.19	1.76	0.44	0.13	0.37	0.30	0.33	0.22
**Median**	101	121	0	0	0	0	3.00	2.00	0	0	0	0	0	0
**Min**	0	0	0	0	0	0	1	0	0	0	0	0	0	0
**Max**	606	1288	1	1	1	1	20	21	1	1	1	1	1	1

	Hotel Style "Luxury"		Room Type "City View"		Room Type "Non-Smoking"		Room Type "Suites"		Room Type "Family"		Room Type "Ocean View"		Room Type "Pool View"	
	GL	Non-GL	GL	Non-GL	GL	GL	GL	Non-GL	GL	Non-GL	GL	Non-GL	GL	Non-GL
**Mean**	0.01	0.01	0.10	0.05	0.95	0.72	0.50	0.28	0.51	0.43	0.13	0.09	0.04	0.02
**Std. Dev.**	0.09	0.08	0.30	0.21	0.21	0.45	0.50	0.45	0.50	0.50	0.34	0.29	0.20	0.14
**Median**	0	0	0	0	1	1	1	0	1	0	0	0	0	0
**Min**	0	0	0	0	0	0	0	0	0	0	0	0	0	0
**Max**	1	1	1	1	1	1	1	1	1	1	1	1	1	1

	Room Type "Bridal"		Amenities Section Length		Amenities "Bicycle"		Amenities "High-Speed Wi-Fi"		Amenities "Restaurant"		Amenities "Paid Parking"		Amenities "Free Parking"	
	GL	Non-GL	GL	Non-GL	GL	GL	GL	Non-GL	GL	Non-GL	GL	Non-GL	GL	Non-GL
**Mean**	0.11	0.07	400	242	0.27	0.18	0.87	0.69	0.63	0.39	0.32	0.14	0.29	0.35
**Std. Dev.**	0.31	0.25	201	190	0.44	0.38	0.34	0.46	0.48	0.49	0.47	0.35	0.46	0.48
**Median**	0	0	380	217	0	0	1	1	1	0	0	0	0	0
**Min**	0	0	6	0	0	0	0	0	0	0	0	0	0	0
**Max**	1	1	1943	1788	1	1	1	1	1	1	1	1	1	1

	Amenities "Fitness"		Amenities "Bar"		Amenities "Children Ent."		Amenities "Pets"		Amenities "Sauna"		Amenities "Hot Tub"		Amenities "Pool"	
	GL	Non-GL	GL	Non-GL	GL	GL	GL	Non-GL	GL	Non-GL	GL	Non-GL	GL	Non-GL
**Mean**	0.38	0.14	0.73	0.48	0.29	0.27	0.47	0.31	0.26	0.13	0.13	0.11	0.30	0.25
**Std. Dev.**	0.49	0.35	0.45	0.50	0.45	0.45	0.50	0.46	0.44	0.34	0.33	0.31	0.46	0.43
**Median**	0	0	1	0	0	0	0	0	0	0	0	0	0	0
**Min**	0	0	0	0	0	0	0	0	0	0	0	0	0	0
**Max**	1	1	1	1	1	1	1	1	1	1	1	1	1	1

	Amenities "Coffee Shop"		Amenities "Special Diet"		Amenities "Breakfast"		Amenities "Breakfast Room"		Amenities "Taxi Service"		Amenities "Airport Service"		Amenities "Business"	
	GL	Non-GL	GL	Non-GL	GL	GL	GL	Non-GL	GL	Non-GL	GL	Non-GL	GL	Non-GL
**Mean**	0.17	0.10	0.19	0.13	0.93	0.63	0.36	0.20	0.26	0.10	0.18	0.17	0.42	0.14
**Std. Dev.**	0.37	0.30	0.39	0.34	0.25	0.48	0.48	0.40	0.44	0.30	0.38	0.37	0.49	0.34
**Median**	0	0	0	0	1	1	0	0	0	0	0	0	0	0
**Min**	0	0	0	0	0	0	0	0	0	0	0	0	0	0
**Max**	1	1	1	1	1	1	1	1	1	1	1	1	1	1

	Amenities “Conference”		Amenities “Banquet”		Amenities “Meeting Rooms”		Amenities “Terrace”		Amenities “Baggage Storage”		Amenities “Concierge”		Amenities “Currency Exch.”	
	GL	Non-GL	GL	Non-GL	GL	GL	GL	Non-GL	GL	Non-GL	GL	Non-GL	GL	Non-GL
**Mean**	0.47	0.14	0.44	0.15	0.59	0.26	0.21	0.18	0.81	0.47	0.48	0.26	0.20	0.08
**Std. Dev.**	0.50	0.35	0.50	0.35	0.49	0.44	0.41	0.39	0.39	0.50	0.50	0.44	0.40	0.27
**Median**	0	0	0	0	1	0	0	0	1	0	0	0	0	0
**Min**	0	0	0	0	0	0	0	0	0	0	0	0	0	0
**Max**	1	1	1	1	1	1	1	1	1	1	1	1	1	1

	Amenities “Non-Smoking”		Amenities “24 Hours”		Amenities “Special Check-In”		Amenities “Dry Cleaning”		Amenities “Landry”		Amenities “Facial Treatment”		Amenities “Spa”	
	GL	Non-GL	GL	Non-GL	GL	GL	GL	Non-GL	GL	Non-GL	GL	Non-GL	GL	Non-GL
**Mean**	0.81	0.65	0.73	0.34	0.35	0.18	0.60	0.25	0.71	0.40	0.11	0.05	0.20	0.12
**Std. Dev.**	0.39	0.48	0.45	0.47	0.48	0.39	0.49	0.43	0.45	0.49	0.31	0.22	0.40	0.33
**Median**	1	1	1	0	0	0	1	0	1	0	0	0	0	0
**Min**	0	0	0	0	0	0	0	0	0	0	0	0	0	0
**Max**	1	1	1	1	1	1	1	1	1	1	1	1	1	1

	Amenities “Massage”		Room Feature Section Length		Room Feature “Allergy Free”		Room Feature “Flatsceen TV”		Room Feature “Walk-In Shower”		Room Feature “Housekeeping”		Room Feature “Coffee & Tea”	
	GL	Non-GL	GL	Non-GL	GL	GL	GL	Non-GL	GL	Non-GL	GL	Non-GL	GL	Non-GL
**Mean**	0.18	0.11	132	124	0.13	0.07	0.93	0.59	0.08	0.05	0.68	0.33	0.16	0.10
**Std. Dev.**	0.39	0.32	94	103	0.33	0.25	0.26	0.49	0.28	0.21	0.47	0.47	0.37	0.31
**Median**	0	0	101	95	0	0	1	1	0	0	1	0	0	0
**Min**	0	0	0	0	0	0	0	0	0	0	0	0	0	0
**Max**	1	1	739	1415	1	1	1	1	1	1	1	1	1	1

	Room Feature “Soundproof”		Room Feature “Safe”		Room Feature “Laptop Safe”		Room Feature “Balcony”		Room Feature “Refrigerator”		Room Feature “Toiletries”		Room Feature “Hairdryer”	
	GL	Non-GL	GL	Non-GL	GL	GL	GL	Non-GL	GL	Non-GL	GL	Non-GL	GL	Non-GL
**Mean**	0.27	0.15	0.71	0.45	0.12	0.04	0.26	0.20	0.24	0.25	0.32	0.26	0.46	0.35
**Std. Dev.**	0.44	0.36	0.45	0.50	0.32	0.20	0.44	0.40	0.43	0.43	0.47	0.44	0.50	0.48
**Median**	0	0	1	0	0	0	0	0	0	0	0	0	0	0
**Min**	0	0	0	0	0	0	0	0	0	0	0	0	0	0
**Max**	1	1	1	1	1	1	1	1	1	1	1	1	1	1

	Room Feature “Fireplace”		Room Feature “Air Conditioning”		Room Feature “Minibar”		Room Feature “Microwave”		Room Feature “VIP Room”		Room Feature “Room Service”		Room Feature “Wake Up Service”	
	GL	Non-GL	GL	Non-GL	GL	GL	GL	Non-GL	GL	Non-GL	GL	Non-GL	GL	Non-GL
**Mean**	0.07	0.03	0.72	0.47	0.37	0.22	0.05	0.08	0.10	0.04	0.52	0.25	0.30	0.17
**Std. Dev.**	0.26	0.18	0.45	0.50	0.48	0.41	0.22	0.26	0.30	0.19	0.50	0.43	0.46	0.38
**Median**	0	0	1	0	0	0	0	0	0	0	1	0	0	0
**Min**	0	0	0	0	0	0	0	0	0	0	0	0	0	0
**Max**	1	1	1	1	1	1	1	1	1	1	1	1	1	1

	Traveler’s Choice Award		Area Comparison		Proportion of English Reviews		Proportion of Local Reviews		GreenLeader Award	
	GL	Non-GL	GL	Non-GL	GL	GL	GL	Non-GL	GL	Non-GL
**Mean**	0.57	0.32	0.71	0.60	0.44	0.40	0.54	0.55	1.00	0.00
**Std. Dev.**	0.50	0.47	0.21	0.27	0.30	0.31	0.31	0.33	0.00	0.00
**Median**	1	0	1	1	0	0	1	1	1	0
**Min**	0	0	0	0	0	0	0	0	1	0
**Max**	1	1	1	1	1	1	1	1	1	0

Supplementary Information IX: Principal Component Analysis

During the exploratory stage, PCA is performed on the set of continuous variables. This subset is chosen to both fulfill the technique's assumptions and because these variables account for the majority of the underlying variation in the data. Performing the same analysis on the whole dataset including binary variables does not change the detected variance structure much and thus offers few additional insights. The variables are scaled using a standard scaler to circumvent the effect of different variable ranges on variation. At this point, data leakage is not an issue, and the scaler is trained with and applied to all available observations. Since PCA is an unsupervised learning technique, no one correct solution for the analysis exists. Accordingly, the number of dimensions to be used is decided using guidelines. The following figure visualizes three guidelines:

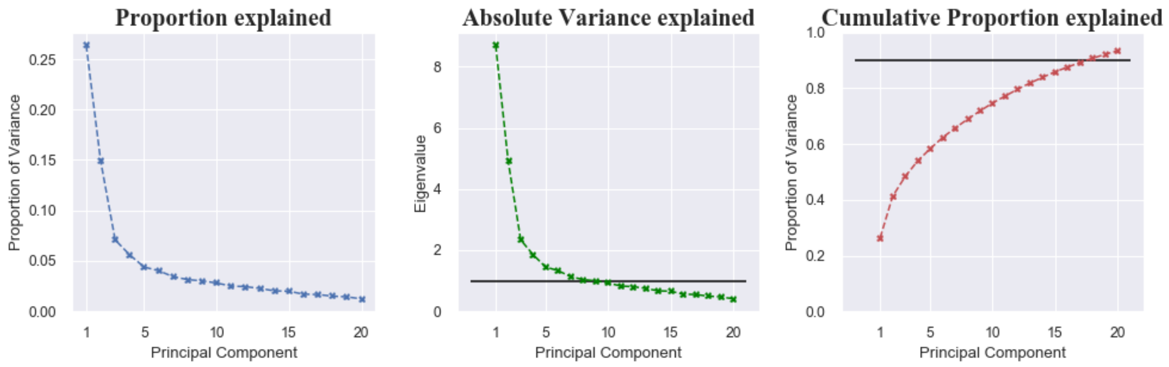

Figure 2: Variance captured by Principal Components

The scree plot on the left visualizes the proportion of total variance the first 20 components capture. While the first PC captures more than 25% of variation and the second PC still captures close to 15%, numbers quickly drop and then fall in a slow but consistent manner. This creates the possibility to determine the optimal number of components via the *elbow rule*. According to this guideline, the optimal number of components for the analysis is four to five. A second guideline, the so-called *Kaiser rule*, advises to include those components with eigenvalues above one. This is visualized via the horizontal line in the second graph. Following the guideline would thus lead to the use of eight components in the further analysis. The final guideline considered is to add components to the analysis until 90% of variation is explained. In the third plot, this threshold is again visualized by a horizontal line. To capture 90% of the variation in the dataset, 18 PCs should be included. Moving forward, the *elbow rule* guideline will be applied during the exploratory stage, where the focus is on getting a first intuition of patterns in the data rather than dimensionality reduction for further use of the data. Other

guidelines will be applied during later stages of the analysis. The largest four principal components are able to capture roughly half of the variation present in the data.

Supplementary Information X: Clustering Analysis

Like PCA, clustering can be used to develop an intuition of patterns in the data. Unlike the above analysis however, the focus of clustering lies in finding groups of similar observations rather than groups of variables. These groups of similar observations, or clusters, can potentially serve to find typical observations or *prototypes*. For the goal at hand, this method of clustering and finding prototypes would ideally inform about differences between groups of sustainable and non-sustainable listings. In the following, the use of PCA as a preprocessing step for the clustering analysis is discussed and applied. Options for the number of clusters are evaluated based on the respective sums of distances and silhouette analysis. Lastly, the clusters determined by the most successful method are reviewed.

To avoid the pitfalls of applying geometric distance measures to high-dimensional data, PCA is applied as a preparatory step for K-means clustering. Due to the dimensionality reduction technique's assumptions, this method is again exclusively applied to continuous variables. Since the unsupervised learning task by definition does not have training labels, the optimal number of clusters is determined with the help of two guidelines. Firstly, a plot showing the sum of distances from all observations to the closest cluster center for K = {1,2,…,10} is analyzed. In a similar fashion to principal component analysis, an appropriate number of clusters can be found via the elbow-method. However, the data is not easily segmented. Both the analysis using the first four and the analysis using the first eight principal components shows no obvious elbow point.

Scree plots for K-means clustering using four and eight PCs

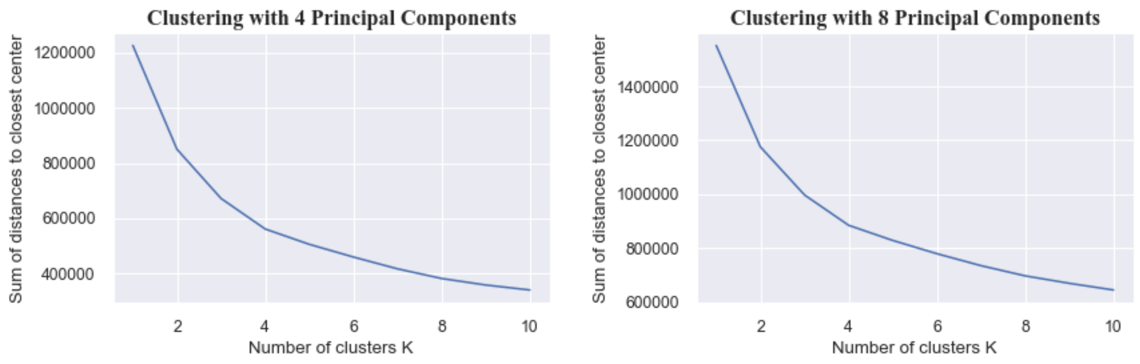

As a second guideline, *Silhouette Analysis* is applied. Like the abovementioned elbow method, this technique employs a distance-based measure to create a numerical basis of comparison for the number of clusters used. In addition to this averaged *Silhouette Score*, a visualization of each single observation's score can aid to better understand groups of observations. The silhouette score is defined as

$$S = \frac{b - a}{max(a, b)}$$

where a is the mean distance between an observation and every other observation in the cluster it is assigned to, referred to as the *mean intra-cluster distance*. b on the other hand measures the *mean nearest-cluster distance*, which averages the distances between an observation and all observations of the closest cluster. The silhouette score returns values between negative one at worst and positive one at best. Intuitively, the cluster assignment is poor when an observation is closer to observations from another cluster than to its peers in the same cluster. Conversely, the more dense and well-separated clusters are, the larger the total average score will be (Kaufman & Rousseeuw, 2009). For K-means clustering of the data's first four principal components, the silhouette plots for $K = \{2,3,4,5\}$ are pictured below. For each K, cluster assignments were calculated with a limit of 100 iterations of the algorithm and selecting the most successful out of ten runs. The diagram shows that the segmentation into two clusters is the only setting in which the average silhouette score, pictured by the dashed red line, is above 0.5. On the other hand, the choice of only two clusters leads to extreme differences in cluster size with a single cluster containing close to 90% of observations. In addition, all observations assigned to the smaller cluster have silhouette scores below the total average. For segmentations into three, four and five clusters, silhouette scores between 0.265 and 0.283 are found. From these very similar and low scores, it can be inferred that clustering of the observations is not an easy task here. The same silhouette analysis was performed for the clustering of observations using the first eight principal components. However, no improvement to the quality of segmentation could be found. Nonetheless, applying clustering to the data can help showcasing structure in the data and identifying *prototype* accommodations.

Silhouette plots for K-means clustering of the largest four PCs

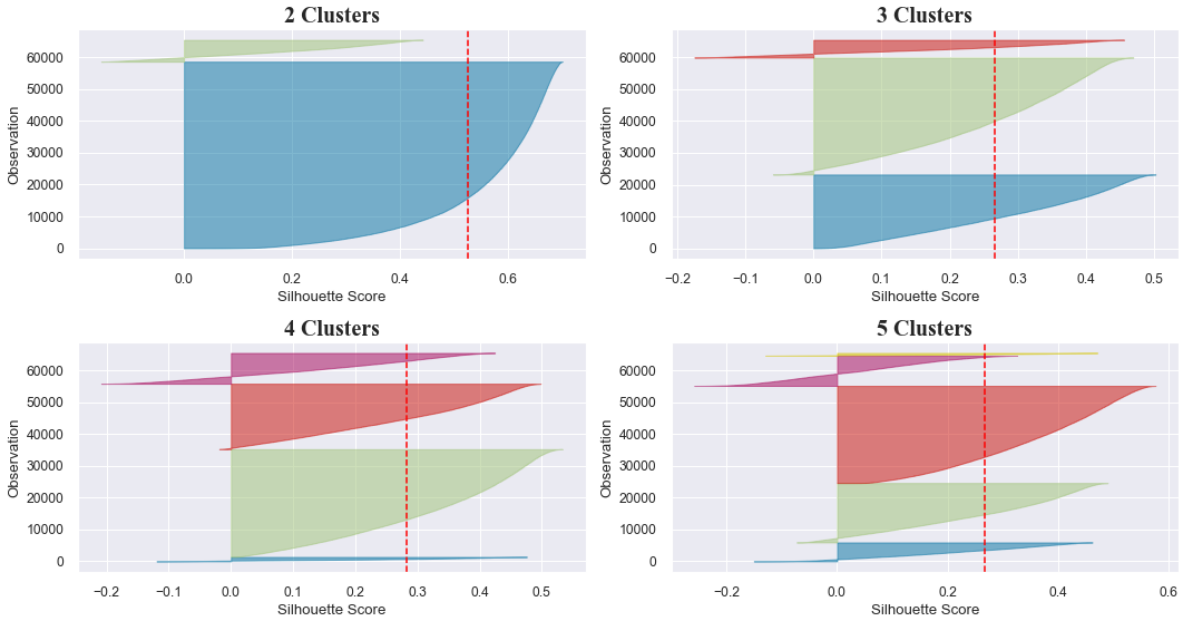

Silhouette plots for K-means clustering of the largest eight PCs

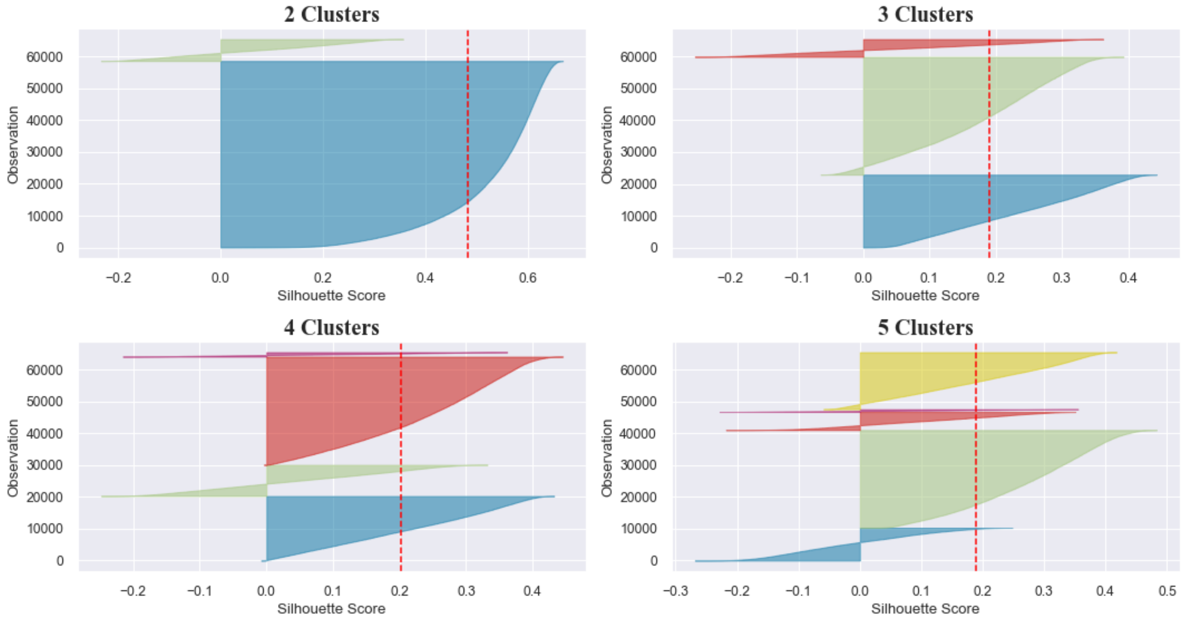

Supplementary Information XI: Classifier performance

LDA without Dimensionality Reduction

	No Transformation			Yeo Johnson			Box Cox			Normalize			Standardize			Robust		
	Recall	F2	ROC	Recall	F2	ROC	Recall	F2	ROC	Recall	F2	ROC	Recall	F2	ROC	Recall	F2	ROC
**None**	0.329	0.331	0.851	0.287	0.299	0.857	0.288	0.3	0.862	0.329	0.331	0.851	0.329	0.331	0.851	0.329	0.331	0.851
**Rand ov.**	0.771	0.382	0.861	0.802	0.397	0.877	0.797	0.396	0.877	0.77	0.381	0.86	0.768	0.381	0.86	0.77	0.381	0.86
**Rand un.**	0.772	0.37	0.857	0.806	0.387	0.875	0.794	0.391	0.875	0.77	0.37	0.856	0.771	0.375	0.859	0.767	0.372	0.857
**SMOTE**	0.025	0.031	0.739	0.786	0.389	0.873	0.791	0.395	0.873	0.741	0.381	0.858	0.752	0.372	0.856	0.752	0.38	0.858
**SMO. un.**	0.025	0.031	0.74	0.787	0.39	0.873	0.791	0.395	0.872	0.741	0.38	0.858	0.755	0.373	0.855	0.752	0.38	0.858

LDA applied to the first four Principal Components

	No Transformation			Yeo Johnson			Box Cox			Normalize			Standardize			Robust		
	Recall	F2	ROC	Recall	F2	ROC	Recall	F2	ROC	Recall	F2	ROC	Recall	F2	ROC	Recall	F2	ROC
**None**	0.157	0.17	0.837	0.001	0.001	0.807	0.001	0.001	0.809	0.001	0.001	0.778	0.108	0.121	0.807	0.118	0.13	0.804
**Rand ov.**	0.651	0.334	0.812	0.747	0.307	0.808	0.733	0.312	0.811	0.71	0.289	0.784	0.703	0.31	0.8	0.539	0.314	0.802
**Rand un.**	0.65	0.333	0.811	0.746	0.306	0.808	0.733	0.312	0.811	0.71	0.289	0.784	0.705	0.312	0.801	0.537	0.313	0.803
**SMOTE**	0.653	0.333	0.812	0.749	0.307	0.808	0.734	0.312	0.811	0.708	0.288	0.784	0.705	0.31	0.801	0.54	0.313	0.802
**SMO. un.**	0.653	0.333	0.812	0.747	0.307	0.808	0.734	0.312	0.811	0.71	0.289	0.784	0.706	0.31	0.8	0.54	0.313	0.802

LDA applied to the first eight Principal Components

	No Transformation			Yeo Johnson			Box Cox			Normalize			Standardize			Robust		
	Recall	F2	ROC	Recall	F2	ROC	Recall	F2	ROC	Recall	F2	ROC	Recall	F2	ROC	Recall	F2	ROC
**None**	0.154	0.167	0.837	0.091	0.106	0.824	0.126	0.144	0.829	0.0	0.0	0.78	0.19	0.203	0.829	0.159	0.171	0.827
**Rand ov.**	0.668	0.33	0.81	0.771	0.335	0.837	0.767	0.342	0.839	0.747	0.288	0.792	0.708	0.339	0.83	0.556	0.345	0.828
**Rand un.**	0.667	0.328	0.809	0.769	0.333	0.837	0.768	0.344	0.839	0.746	0.288	0.792	0.708	0.339	0.83	0.557	0.345	0.829
**SMOTE**	0.668	0.329	0.81	0.77	0.336	0.837	0.765	0.342	0.839	0.75	0.29	0.792	0.706	0.341	0.83	0.566	0.349	0.825
**SMO. un.**	0.669	0.329	0.81	0.768	0.334	0.837	0.765	0.343	0.839	0.75	0.289	0.792	0.708	0.341	0.83	0.567	0.35	0.825

QDA without Dimensionality Reduction

	No Transformation			Yeo Johnson			Box Cox			Normalize			Standardize			Robust		
	Recall	F2	ROC	Recall	F2	ROC	Recall	F2	ROC	Recall	F2	ROC	Recall	F2	ROC	Recall	F2	ROC
**None**	0.568	0.356	0.82	0.715	0.367	0.852	0.722	0.376	0.857	0.661	0.376	0.846	0.662	0.374	0.846	0.657	0.377	0.844
**Rand ov.**	0.615	0.347	0.822	0.777	0.353	0.852	0.778	0.36	0.857	0.71	0.368	0.846	0.711	0.366	0.845	0.691	0.369	0.835
**Rand un.**	0.867	0.214	0.715	0.456	0.317	0.846	0.373	0.285	0.852	0.515	0.316	0.839	0.533	0.355	0.84	0.578	0.32	0.813
**SMOTE**	0.415	0.324	0.697	0.703	0.394	0.85	0.698	0.4	0.854	0.708	0.401	0.845	0.699	0.399	0.845	0.68	0.401	0.835
**SMO. un.**	0.412	0.32	0.697	0.705	0.394	0.85	0.7	0.403	0.853	0.707	0.398	0.846	0.699	0.4	0.846	0.679	0.404	0.834

QDA with PCA with 4 Dimensions

	No Transformation			Yeo Johnson			Box Cox			Normalize			Standardize			Robust		
	Recall	F2	ROC	Recall	F2	ROC	Recall	F2	ROC	Recall	F2	ROC	Recall	F2	ROC	Recall	F2	ROC
**None**	0.243	0.228	0.814	0.025	0.03	0.804	0.022	0.027	0.807	0.094	0.107	0.784	0.155	0.162	0.788	0.163	0.159	0.769
**Rand ov.**	0.445	0.32	0.814	0.753	0.301	0.804	0.739	0.311	0.807	0.762	0.276	0.785	0.614	0.311	0.788	0.3	0.243	0.768
**Rand un.**	0.447	0.321	0.814	0.757	0.302	0.804	0.742	0.311	0.807	0.756	0.275	0.785	0.611	0.309	0.788	0.303	0.242	0.77
**SMOTE**	0.458	0.332	0.816	0.75	0.3	0.804	0.735	0.31	0.807	0.765	0.277	0.784	0.622	0.311	0.79	0.309	0.247	0.776
**SMO. un.**	0.457	0.331	0.816	0.751	0.3	0.804	0.734	0.31	0.807	0.762	0.275	0.784	0.624	0.312	0.79	0.308	0.246	0.776

QDA with PCA with 8 Dimensions

	No Transformation			Yeo Johnson			Box Cox			Normalize			Standardize			Robust		
	Recall	F2	ROC	Recall	F2	ROC	Recall	F2	ROC	Recall	F2	ROC	Recall	F2	ROC	Recall	F2	ROC
**None**	0.28	0.249	0.809	0.286	0.263	0.828	0.301	0.274	0.831	0.171	0.175	0.801	0.311	0.273	0.813	0.215	0.198	0.774
**Rand ov.**	0.459	0.315	0.809	0.814	0.311	0.828	0.805	0.321	0.831	0.823	0.275	0.8	0.703	0.335	0.813	0.338	0.261	0.774
**Rand un.**	0.47	0.314	0.805	0.819	0.312	0.828	0.807	0.319	0.83	0.822	0.275	0.799	0.7	0.332	0.81	0.342	0.258	0.769
**SMOTE**	0.506	0.337	0.8	0.804	0.313	0.829	0.797	0.324	0.832	0.809	0.277	0.801	0.713	0.332	0.814	0.354	0.274	0.77
**SMO. un.**	0.501	0.335	0.8	0.806	0.313	0.829	0.797	0.324	0.832	0.81	0.277	0.801	0.712	0.331	0.814	0.353	0.273	0.77

Logistic Regression without Dimensionality Reduction

	No Transformation			Yeo Johnson			Box Cox			Normalize			Standardize			Robust		
	Recall	F2	ROC	Recall	F2	ROC	Recall	F2	ROC	Recall	F2	ROC	Recall	F2	ROC	Recall	F2	ROC
**None**	0.04	0.048	0.798	0.108	0.128	0.876	0.106	0.126	0.875	0.097	0.115	0.858	0.1	0.118	0.858	0.095	0.112	0.852
**Rand ov.**	0.706	0.372	0.838	0.795	0.399	0.878	0.8	0.401	0.878	0.761	0.387	0.865	0.76	0.391	0.866	0.764	0.386	0.865
**Rand un.**	0.708	0.372	0.837	0.802	0.391	0.875	0.802	0.394	0.876	0.766	0.371	0.859	0.767	0.383	0.862	0.773	0.379	0.861
**SMOTE**	0.635	0.347	0.807	0.779	0.395	0.874	0.783	0.398	0.874	0.733	0.386	0.861	0.741	0.383	0.86	0.742	0.383	0.861
**SMO. un.**	0.654	0.351	0.809	0.776	0.393	0.874	0.784	0.399	0.874	0.734	0.387	0.861	0.738	0.381	0.86	0.748	0.383	0.861

Logistic Regression with PCA with 4 Dimensions

	No Transformation			Yeo Johnson			Box Cox			Normalize			Standardize			Robust		
	Recall	F2	ROC	Recall	F2	ROC	Recall	F2	ROC	Recall	F2	ROC	Recall	F2	ROC	Recall	F2	ROC
**None**	0.029	0.035	0.823	0.0	0.001	0.807	0.0	0.001	0.81	0.0	0.0	0.784	0.014	0.018	0.801	0.024	0.029	0.801
**Rand ov.**	0.673	0.36	0.834	0.744	0.308	0.808	0.736	0.312	0.811	0.713	0.287	0.784	0.704	0.315	0.805	0.608	0.318	0.805
**Rand un.**	0.677	0.36	0.834	0.745	0.307	0.808	0.737	0.312	0.811	0.717	0.288	0.783	0.706	0.316	0.805	0.607	0.316	0.804
**SMOTE**	0.674	0.359	0.833	0.744	0.308	0.808	0.735	0.311	0.811	0.715	0.288	0.784	0.708	0.315	0.805	0.607	0.317	0.805
**SMO. un.**	0.675	0.359	0.833	0.743	0.308	0.808	0.735	0.312	0.811	0.716	0.289	0.784	0.706	0.315	0.805	0.607	0.317	0.805

Logistic Regression with PCA with 8 Dimensions

	No Transformation			Yeo Johnson			Box Cox			Normalize			Standardize			Robust		
	Recall	F2	ROC	Recall	F2	ROC	Recall	F2	ROC	Recall	F2	ROC	Recall	F2	ROC	Recall	F2	ROC
**None**	0.025	0.031	0.817	0.007	0.009	0.836	0.006	0.007	0.839	0.0	0.0	0.792	0.031	0.038	0.83	0.035	0.043	0.824
**Rand ov.**	0.681	0.359	0.834	0.768	0.335	0.837	0.768	0.342	0.839	0.747	0.289	0.792	0.731	0.351	0.838	0.646	0.354	0.83
**Rand un.**	0.686	0.359	0.834	0.768	0.335	0.837	0.766	0.342	0.839	0.747	0.29	0.792	0.726	0.349	0.837	0.644	0.353	0.829
**SMOTE**	0.685	0.358	0.831	0.763	0.335	0.836	0.763	0.342	0.839	0.747	0.29	0.792	0.728	0.348	0.837	0.652	0.357	0.827
**SMO. un.**	0.684	0.358	0.831	0.763	0.335	0.836	0.763	0.342	0.839	0.747	0.29	0.792	0.728	0.349	0.837	0.652	0.357	0.827

Random Forest without Dimensionality Reduction

	No Transformation			Yeo Johnson			Box Cox			Normalize			Standardize			Robust		
	Recall	F2	ROC	Recall	F2	ROC	Recall	F2	ROC	Recall	F2	ROC	Recall	F2	ROC	Recall	F2	ROC
**None**	0.066	0.08	0.877	0.065	0.079	0.877	0.063	0.077	0.876	0.061	0.074	0.877	0.062	0.076	0.879	0.061	0.074	0.876
**Rand ov.**	0.15	0.176	0.88	0.144	0.17	0.88	0.14	0.165	0.879	0.147	0.173	0.883	0.143	0.168	0.881	0.148	0.174	0.882
**Rand un.**	0.803	0.395	0.881	0.798	0.392	0.88	0.797	0.397	0.883	0.801	0.397	0.883	0.796	0.394	0.882	0.8	0.396	0.882
**SMOTE**	0.222	0.247	0.875	0.231	0.26	0.886	0.216	0.245	0.886	0.256	0.283	0.887	0.22	0.249	0.886	0.169	0.197	0.885
**SMO. un.**	0.218	0.244	0.874	0.23	0.259	0.885	0.216	0.244	0.883	0.263	0.291	0.884	0.219	0.249	0.883	0.165	0.192	0.882

Random Forest with PCA with 4 Dimensions

	No Transformation			Yeo Johnson			Box Cox			Normalize			Standardize			Robust		
	Recall	F2	ROC	Recall	F2	ROC	Recall	F2	ROC	Recall	F2	ROC	Recall	F2	ROC	Recall	F2	ROC
**None**	0.048	0.058	0.817	0.016	0.02	0.778	0.007	0.008	0.784	0.006	0.008	0.749	0.016	0.02	0.775	0.005	0.006	0.755
**Rand ov.**	0.091	0.105	0.807	0.052	0.061	0.769	0.042	0.05	0.774	0.026	0.032	0.74	0.056	0.066	0.777	0.032	0.038	0.75
**Rand un.**	0.76	0.344	0.84	0.73	0.304	0.806	0.744	0.311	0.81	0.712	0.278	0.776	0.734	0.312	0.81	0.739	0.293	0.788
**SMOTE**	0.425	0.324	0.809	0.331	0.254	0.774	0.324	0.249	0.774	0.281	0.217	0.739	0.338	0.261	0.777	0.311	0.224	0.753
**SMO. un.**	0.43	0.33	0.81	0.329	0.254	0.771	0.324	0.25	0.774	0.285	0.219	0.737	0.343	0.264	0.778	0.31	0.224	0.752

Random Forest with PCA with 8 Dimensions

	No Transformation			Yeo Johnson			Box Cox			Normalize			Standardize			Robust		
	Recall	F2	ROC	Recall	F2	ROC	Recall	F2	ROC	Recall	F2	ROC	Recall	F2	ROC	Recall	F2	ROC
**None**	0.03	0.037	0.836	0.024	0.029	0.835	0.02	0.024	0.83	0.003	0.004	0.794	0.041	0.051	0.834	0.016	0.02	0.822
**Rand ov.**	0.079	0.094	0.834	0.059	0.071	0.835	0.058	0.07	0.836	0.03	0.037	0.793	0.077	0.092	0.837	0.053	0.063	0.822
**Rand un.**	0.775	0.352	0.851	0.778	0.355	0.848	0.781	0.352	0.849	0.733	0.309	0.811	0.776	0.361	0.851	0.769	0.34	0.839
**SMOTE**	0.412	0.354	0.842	0.373	0.331	0.84	0.382	0.339	0.837	0.293	0.265	0.797	0.401	0.354	0.84	0.385	0.325	0.825
**SMO. un.**	0.414	0.357	0.842	0.374	0.331	0.839	0.378	0.336	0.839	0.3	0.271	0.797	0.396	0.349	0.839	0.38	0.322	0.824

Supplementary Information XII: Estimated proportion of GreenLeaders by country

**Country**	**Proportion of GreenLeaders**	
	In Training Data	Estimated (QDA)
Austria	0.025	0.085
Belgium	0.085	0.190
Bulgaria	/	0.043
Croatia	/	0.070
Cyprus	/	0.223
Czech Republic	/	0.094
Denmark	0.036	0.259
England	0.041	0.178
Estonia	/	0.169
Finland	0.075	0.289
France	0.057	0.184
Germany	0.040	0.129
Greece	0.012	0.061
Hungary	/	0.139
Iceland	0.055	0.181
Ireland	0.039	0.297
Italy	0.023	0.088
Latvia	/	0.125
Liechtenstein	/	0.000
Lithuania	/	0.076
Luxembourg	0.136	0.237
Malta	/	0.223
Monaco	0.455	0.545
Netherlands	0.081	0.221
Northern Ireland	0.054	0.304
Norway	0.053	0.294
Poland	/	0.094
Portugal	0.037	0.178
Romania	/	0.041
San Marino	/	0.25
Scotland	0.039	0.170
Slovakia	/	0.046
Slovenia	/	0.08
Spain	0.035	0.179
Sweden	0.047	0.284
Switzerland	0.056	0.154
Wales	0.041	0.113
**Total**	**0.036**	**0.136**

Literature

Abdi, H., & Williams, L. J. (2010). Principal component analysis. *Wiley Interdisciplinary Reviews: Computational Statistics*, *2*(4), 433–459.

Aguinis, H., Gottfredson, R. K., & Joo, H. (2013). Best-Practice Recommendations for Defining, Identifying, and Handling Outliers. *Organizational Research Methods*, *16*(2), 270–301. https://doi.org/10.1177/1094428112470848

Askitas, N., & Zimmermann, K. F. (2009). Google Econometrics and Unemployment Forecasting. *Applied Economics Quarterly*, *55*(2), 107–120. https://doi.org/10.3790/aeq.55.2.107

Bellotti, T., & Crook, J. (2009). Support vector machines for credit scoring and discovery of significant features. *Expert Systems with Applications*, *36*(2), 3302–3308. https://doi.org/10.1016/j.eswa.2008.01.005

Bishop, C. M. (2006). *Pattern recognition and machine learning*. Springer.

Box, G. E., & Cox, D. R. (1964). An analysis of transformations. *Journal of the Royal Statistical Society: Series B (Methodological)*, *26*(2), 211–243.

Braesemann, F. (2018). *Estimating the Number of Jobs in Sustainable Tourism in Albania using Big Social Data from TripAdvisor and Booking.com* [Project Report]. https://drive.google.com/open?id=1OwNOEHlBcMiEcoR0jZ_IR6erEz47trzl

Branco, P., Torgo, L., & Ribeiro, R. P. (2016). A Survey of Predictive Modeling on Imbalanced Domains. *ACM Computing Surveys*, *49*(2), 1–50. https://doi.org/10.1145/2907070

Brownlee, J. (2016). *Machine learning mastery with Python: Understand your data, create accurate models, and work projects end-to-end*. Machine Learning Mastery.

Brownlee, J. (2020). *Imbalanced classification with Python: Better metrics, balance skewed classes, cost-sensitive learning*. Machine Learning Mastery.

Byrd, R. H., Lu, P., Nocedal, J., & Zhu, C. (1995). A limited memory algorithm for bound constrained optimization. *SIAM Journal on Scientific Computing*, *16*(5), 1190–1208.

Carrière-Swallow, Y., & Labbé, F. (2013). Nowcasting with Google Trends in an Emerging Market: Nowcasting with Google Trends in an Emerging Market. *Journal of Forecasting*, *32*(4), 289–298. https://doi.org/10.1002/for.1252

Chawla, N. V., Bowyer, K. W., Hall, L. O., & Kegelmeyer, W. P. (2002). SMOTE: synthetic minority over-sampling technique. *Journal of Artificial Intelligence Research*, *16*, 321–357.

Choi, H., & Varian, H. (2009). Predicting initial claims for unemployment benefits. *Google Inc*, *1*, 1–5.

Data-Pop Alliance. (2015, October 21). *Vision and Members*. Data-Pop Alliance. https://datapopalliance.org/about/vision-and-members/

European Commission, & Directorate-General for Internal Market, I., Entrepreneurship and SMEs. (2016). *The European tourism indicator system: ETIS toolkit for sustainable destination management.* Publications Office. https://ec.europa.eu/docsroom/documents/21749

Fatehkia, M., Kashyap, R., & Weber, I. (2018). Using Facebook ad data to track the global digital gender gap. *World Development*, *107*, 189–209. https://doi.org/10.1016/j.worlddev.2018.03.007

Fernández, A., García, S., Galar, M., Prati, R. C., Krawczyk, B., & Herrera, F. (2018). *Learning from imbalanced data sets* (Vol. 10). Springer.

GSTC. (2021). *The GSTC Criteria and the UN SDGs*. Global Sustainable Tourism Council (GSTC). https://www.gstcouncil.org/gstc-criteria/gstc-and-sdgs/

Hair, J. F., Black, W. C., Babin, B. J., & Anderson, R. E. (2019). *Multivariate data analysis* (Eighth edition). Cengage.

Hastie, T., Tibshirani, R., & Friedman, J. H. (2009). *The elements of statistical learning: Data mining, inference, and prediction* (2nd ed). Springer.

He, H., & Ma, Y. (2013). *Imbalanced learning foundations, algorithms, and applications*. John Wiley & Sons, Inc. http://onlinelibrary.wiley.com/book/10.1002/9781118646106

Hilbert, M. (2016). Big Data for Development: A Review of Promises and Challenges. *Development Policy Review*, *34*(1), 135–174. https://doi.org/10.1111/dpr.12142

IAEG-SDGs. (2020). *Tier Classification for Global SDG Indicators as of 17 July 2020*. Inter-agency and Expert Group on SDG Indicators (IAEG-SDGs). https://unstats.un.org/sdgs/files/Tier%20Classification%20of%20SDG%20Indicators_17%20July%202020_web.v2.pdf

James, G., Witten, D., Hastie, T., & Tibshirani, R. (Eds.). (2013). *An introduction to statistical learning: With applications in R*. Springer.

Kaufman, L., & Rousseeuw, P. J. (2009). *Finding Groups in Data: An Introduction to Cluster Analysis*. John Wiley & Sons.

Kirkpatrick, R., & Vacarelu, F. (2019). A decade of leveraging big data for sustainable development. *UN Chronicle*, *55*(4), 26–31.

Lazer, D., Kennedy, R., King, G., & Vespignani, A. (2014). The parable of Google Flu: Traps in big data analysis. *Science*, *343*(6176), 1203–1205.

Le Blanc, D. (2015). Towards Integration at Last? The Sustainable Development Goals as a Network of Targets: The sustainable development goals as a network of targets. *Sustainable Development*, *23*(3), 176–187. https://doi.org/10.1002/sd.1582

Lemaître, G., Nogueira, F., & Aridas, C. K. (2017). Imbalanced-learn: A python toolbox to tackle the curse of imbalanced datasets in machine learning. *The Journal of Machine Learning Research*, *18*(1), 559–563.

Lenzen, M., Sun, Y.-Y., Faturay, F., Ting, Y.-P., Geschke, A., & Malik, A. (2018). The carbon footprint of global tourism. *Nature Climate Change*, *8*(6), 522–528. https://doi.org/10.1038/s41558-018-0141-x

Letouzé, E., Stock, M., Chiara, F., Lizzi, A., & Mazariegos, C. (2019). *Harnessing Innovative Data and Technology to Measure Development Effectiveness*. Southern Voice. http://southernvoice.org/wp-content/uploads/2019/07/190528-Occasional-Paper-Series-54_final.pdf

Mahalanobis, P. C. (1936). *On the generalized distance in statistics*.

Modica, P., Capocchi, A., Foroni, I., & Zenga, M. (2018). An Assessment of the Implementation of the European Tourism Indicator System for Sustainable Destinations in Italy. *Sustainability*, *10*(9), 3160. https://doi.org/10.3390/su10093160

Naef, E., Muelbert, P., Raza, S., Frederick, R., Kendall, J., & Gupta, N. (2014). Using mobile data for development. *Cartesian and Bill and Melinda Gates Foundation, Boston and Seattle*.

Nelson, N. P., Brownstein, J. S., & Hartley, D. M. (2010). Event-based biosurveillance of respiratory disease in Mexico, 2007–2009: Connection to the 2009 influenza A(H1N1) pandemic? *Eurosurveillance*, *15*(30). https://doi.org/10.2807/ese.15.30.19626-en

OECD. (2020). *Rebuilding tourism for the future: COVID-19 policy responses and recovery* (OECD Policy Responses to Coronavirus (COVID-19)) [OECD Policy Responses to Coronavirus (COVID-19)]. https://doi.org/10.1787/bced9859-en

Pedregosa, F., Varoquaux, G., Gramfort, A., Michel, V., Thirion, B., Grisel, O., Blondel, M., Prettenhofer, P., Weiss, R., Dubourg, V., & others. (2011). Scikit-learn: Machine learning in Python. *The Journal of Machine Learning Research*, *12*, 2825–2830.

Plüss, C., Zotz, A., Monshausen, A., & Kühhas, C. (2012). *Sustainability in tourism: A guide through the label jungle*. Naturefriends International. https://www.tourism-watch.de/system/files/migrated/labelguide_en_web.pdf

Rasoolimanesh, S. M., Ramakrishna, S., Hall, C. M., Esfandiar, K., & Seyfi, S. (2020). A systematic scoping review of sustainable tourism indicators in relation to the sustainable development goals. *Journal of Sustainable Tourism*, *0*(0), 1–21. https://doi.org/10.1080/09669582.2020.1775621

Salah, A. A., Pentland, A., Lepri, B., & Letouzé, E. (2019). *Guide to Mobile Data Analytics in Refugee Scenarios*. Springer.

Schwarz, B., Pestre, G., Tellman, B., Sullivan, J., Kuhn, C., Mahtta, R., Pandey, B., & Hammett, L. (2018). Mapping floods and assessing flood vulnerability for disaster decision-making: A case study remote sensing application in Senegal. In *Earth observation open science and innovation* (pp. 293–300). Springer, Cham.

Statistisches Bundesamt. (2021). *Geöffnete Beherbergungsbetriebe, angebotene Schlafgelegenheiten: Stand Juli.* Statistisches Bundesamt (Destatis). https://www-genesis.destatis.de/genesis/online?sequenz=tabelleErgebnis&selectionname=45412-0022

Steele, J. E., Sundsøy, P. R., Pezzulo, C., Alegana, V. A., Bird, T. J., Blumenstock, J., Bjelland, J., Engø-Monsen, K., de Montjoye, Y.-A., Iqbal, A. M., Hadiuzzaman, K. N., Lu, X., Wetter, E., Tatem, A. J., & Bengtsson, L. (2017). Mapping poverty using mobile phone and satellite data. *Journal of The Royal Society Interface*, *14*(127), 20160690. https://doi.org/10.1098/rsif.2016.0690

Stock, J. H., & Watson, M. W. (2012). *Introduction to econometrics* (3. ed., global ed., internat. ed). Pearson.

Teubner, T., & Glaser, F. (2018). Up or out—The dynamics of star rating scores on Airbnb. *Research Papers*. https://aisel.aisnet.org/ecis2018_rp/96

Tingzon, I., Orden, A., Sy, S., Sekara, V., Weber, I., Fatehkia, M., Herranz, M. G., & Kim, D. (2019). Mapping poverty in the Philippines using machine learning, satellite imagery, and crowd-sourced geospatial information. *AI for Social Good ICML 2019 Workshop*.

UNDP & UN Global Pulse. (2016). *A Guide to Data Innovation for Development—From idea to proof-of-concept*. United Nations Development Programme (UNDP). https://www.undp.org/publications/guide-data-innovation-development-idea-proof-concept

United Nations. (2015). *Transforming our world: The 2030 Agenda for Sustainable Development*. https://sdgs.un.org/2030agenda

United Nations. (2020). *The Sustainable Development Goals Report 2020*. United Nations.

Van Belle, G., & Fisher, L. (Eds.). (2004). *Biostatistics: A methodology for the health sciences* (2nd ed). John Wiley & Sons.

Verbraucher Initiative e.V. (2021). *Tripadvisor Green Leaders: Öko-Spitzenreiter*. LABEL-ONLINE: Das Portal Mit Informationen Und Bewertungen Zu Labeln in Deutschland. https://label-online.de/label/tripadvisor-green-leaders-oeko-spitzenreiter/

World Tourism Organization (Ed.). (2004). *Indicators of sustainable development for tourism destinations: A guidebook*. WTO.

World Tourism Organization (Ed.). (2021). *International Tourism Highlights, 2020 Edition*. World Tourism Organization (UNWTO). https://doi.org/10.18111/9789284422456

World Tourism Organization & United Nations Development Programme. (2017). *Tourism and the sustainable development goals: Journey to 2030.*

World Tourism Organization (UNWTO) & International Transport Forum (Eds.). (2019). *Transport-related CO2 Emissions of the Tourism Sector – Modelling Results*. World Tourism Organization (UNWTO). https://doi.org/10.18111/9789284416660

World Travel & Tourism Council. (2021). *Economic Impact Reports*. World Travel & Tourism Council. https://wttc.org/Research/Economic-Impact

Yeo, I.-K., & Johnson, R. A. (2000). A new family of power transformations to improve normality or symmetry. *Biometrika*, *87*(4), 954–959.
